# Conjugate of ibrutinib with a TLR7 agonist suppresses melanoma progression and enhances antitumor immunity

**DOI:** 10.7150/ijbs.64094

**Published:** 2022-01-01

**Authors:** Sumei Ren, Xiaodong Wang, Guangyi Jin

**Affiliations:** 1 School of Pharmaceutical Sciences, Nation-Regional Engineering Lab for Synthetic Biology of Medicine, International Cancer Center, Shenzhen University Health Science Center, Shenzhen University, Shenzhen 518060, Guangdong, China.; 2 Key Laboratory of Optoelectronic Devices and Systems of Ministry of Education and Guangdong Province, College of Optoelectronic Engineering, Shenzhen University, Shenzhen 518060, China.

**Keywords:** ibrutinib, TLR7 agonist, melanoma, immunotherapy

## Abstract

The use of large molecules for immunotherapy has led to exciting developments in cancer treatment, such as the development of PD-1/PD-L1 antibodies. However, small molecule targeted therapies still lack effective immune-functional classes. Ideal anticancer drugs should simultaneously generate immune memory when killing cancer cells to prevent tumor relapse and metastasis. To this end, we carried out a rationally designed strategy to develop novel classes of small molecule compounds with bifunctional targeting and immunostimulatory abilities by conjugating targeting compounds with TLR7 agonists, generating immune-targeting conjugates (ImmunTacs). GY161, as a representative ImmunTac, was synthesized via chemical conjugation of ibrutinib with a TLR7 agonist. *In vitro*, GY161 stimulated the production of cytokines by mouse spleen lymphocytes, promoted the maturation of dendritic cells (DCs), and inhibited the growth and induced the apoptosis of B16 melanoma cells by regulating the c-Met/β-catenin pathway. *In vivo*, GY161 enhanced the frequency of CD8^+^ T cells in spleens and tumors, suppressed the growth of B16 melanoma cell-derived tumors and prolonged the survival time of mice. In summary, GY161 could prevent melanoma progression through direct tumor killing and by triggering specific immunity. These results strongly suggest that ImmunTacs are a reliable and promising strategy for developing small molecule immunogenic anticancer drugs.

## Introduction

Bruton's tyrosine kinase (BTK) is a key kinase in the B cell receptor (BCR) signal transduction pathway and is widely expressed in different types of hematological malignancy cells 1. BTK is also expressed by other immune cells, including myeloid-derived suppressor cells (MDSCs) and tumor-associated macrophages (TAMs), and its immune regulatory effect on myeloid cells has an important impact on the tumor microenvironment (TME) 2. Inhibition of BTK promotes the differentiation of MDSCs into mature DCs and reverses the suppression of antitumor immunity 3, 4. BTK inhibitors (BTKi) can also promote the proliferation and activation of T cells and target ATM to inhibit the production of chemokines (CXCL12 and CXCL13) and angiogenic cytokines 3, 5. Therefore, BTKi can be used for the treatment of tumors other than B cell malignancies. The beneficial effects of BTKi on the immune microenvironment are promising for treating even solid tumors that do not express BTK. Ibrutinib, a first-in-class irreversible BTK inhibitor approved for the treatment of chronic B cell malignancies, has great potential therapeutic value in a variety of solid tumors 2, 6, 7.

Toll-like receptors (TLRs) play an important role in innate and adaptive immunity by recognizing pathogen-associated molecular patterns (PAMPs) present in a variety of viruses and microorganisms 8-11. Among these receptors, TLR7, which recognizes single-stranded RNA and synthetic agonists, is expressed by plasmacytoid dendritic cells (pDCs), macrophages and B lymphocytes 12, 13. TLR7 agonists show strong immunostimulatory activity, inducing tumor-specific immune responses and reducing the growth of colon, renal, and breast carcinomas and melanoma 14-16. Our laboratory has been committed to the development of TLR7 agonists to activate the innate immune system against cancer. We have previously reported the synthesis of a TLR7 agonist, SZU-101, and its use in combination with doxorubicin and in the form of chemical conjugation with JQ-1 for cancer therapy 17, 18.

To synergize their effects, we conjugated ibrutinib with a TLR7 agonist to prepare an immune-targeting conjugate (ImmunTac). The ImmunTac released tumor antigens from killed tumor cells. The antigens were then transferred to and recognized by DCs through interactions between TLR7 agonists and TLR7. The DCs were stimulated and matured in order to present antigens to CD8 and CD4 T cells and B cells, and memory cytotoxic T lymphocytes (CTLs) and antibodies were generated. Moreover, the conjugation of ibrutinib with a TLR7 agonist possibly reversed the immunosuppressive nature of the TME by targeting MDSCs or TAMs. GY161, a representative ImmunTac, was synthesized via chemical conjugation of ibrutinib with the TLR7 agonist GY100. In this study, we found that GY161 regulated the c-Met/β-catenin pathway in B16 tumors and induced tumor-specific immune responses. In the B16 melanoma tumor model, administration of GY161 elicited strong antitumor effects. Our innovative work presented here will provide a new mechanism by which to identify novel immunogenic and tumoricidal small molecule drugs.

## Materials and Methods

### Compounds

**Scheme 1:**
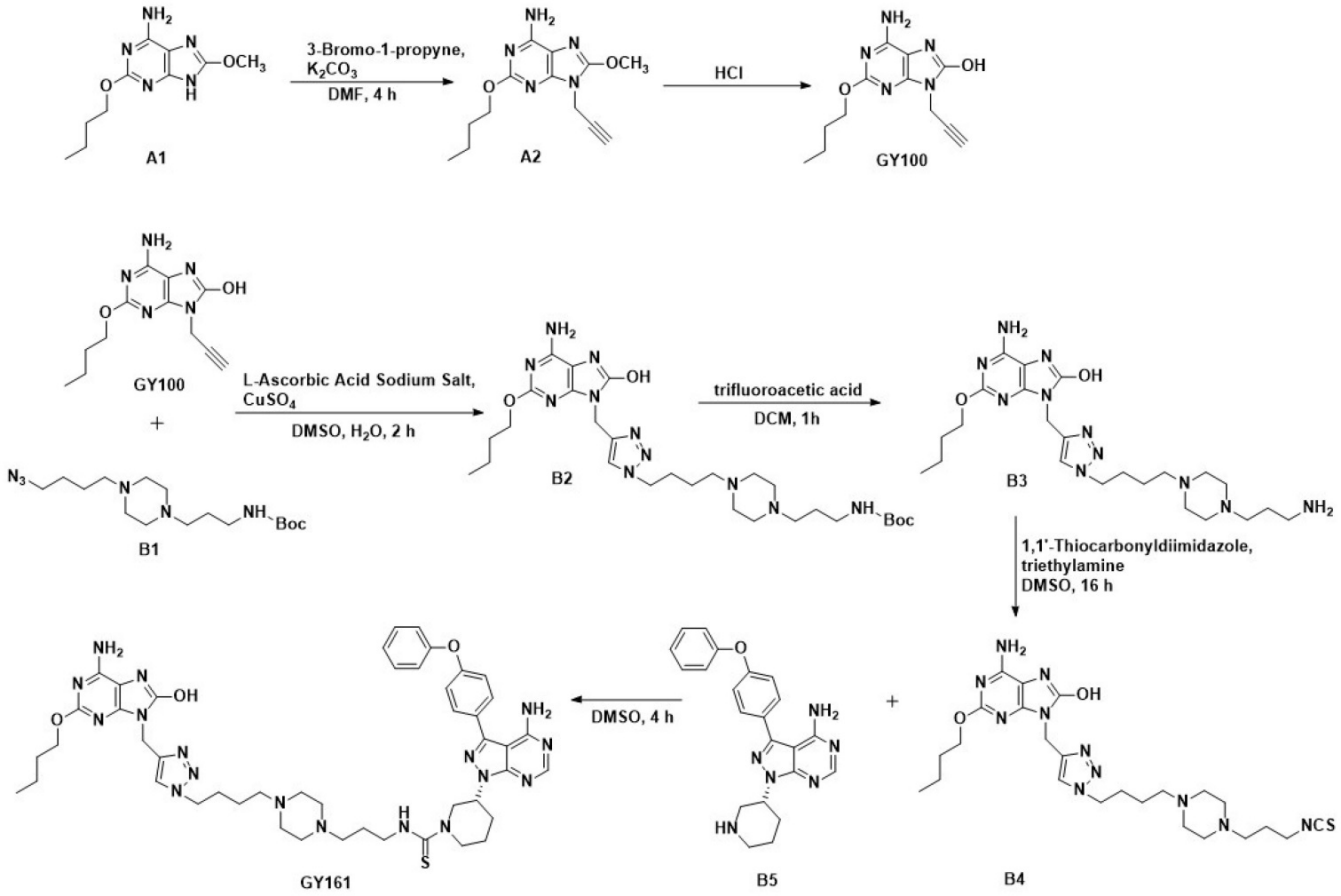
Route of GY100 and GY161 synthesis.

Ibrutinib was purchased from Bide Pharmatech, Ltd. (Shanghai, China). The procedures for synthesizing the target compounds are shown in Scheme 1 (synthesis method in the Supplementary materials).

### Cell culture

B16-F10 (B16) (mouse melanoma cell line), A375 (human melanoma cell line), DaudiB (human B cell lymphoma line), A20 (mouse B cell lymphoma line) and MCF-10A (human normal breast cell line) cells were purchased from ATCC (Manassas, USA). B16 and A375 cells were cultured in DMEM (cat. no. C11995500BT; Gibco, Grand Island, USA) supplemented with 10% FBS (cat. no. A31608; Gibco). DaudiB and A20 cells were cultured in RPMI 1640 medium (cat. no. 01-100-1A; BI, Kibbutz Beit Haemek, Israel) with 10% FBS. MCF-10A cells were cultured in complete medium (Procell, Wuhan, China).

### HEK-Blue assay

HEK-Blue hTLR7 and hTLR8 cells (InvivoGen, San Diego, USA) were cultured in DMEM supplemented with 10% FBS, blasticidin (cat. no. ant-bl-05; InvivoGen, San Diego, USA), zeocin (cat. no. ant-zn-05; InvivoGen) and normocin (cat. no. ant-nr-1; InvivoGen). The cells (2.5×10^4^/200 µL/well) were seeded into 96-well plates and then treated with compounds (0.1, 1.0, 5.0, 10.0, 20.0 and 40.0 μM) in HEK-Blue Detection medium (cat. no. hb-det3; InvivoGen) for 14 h. The activation of TLR7 and TLR8 was assessed by an Automatic microplate reader (BioTek, Vermont, USA).

### Cytokine assay

Lymphocytes isolated from C57BL/6J mice were seeded into 24-well plates at a density of 1×10^6^ cells/mL/well in RPMI 1640 medium supplemented with 10% FBS. The cells were treated for 24 h with the compounds. IL-6, IL-12p70, TNF-α and IFN-γ secretion into the cell culture supernatants was assessed by ELISA kits (88-7064-88, 88-7121-88, 88-7324-88 and 88-7314-88; Invitrogen, Waltham, USA).

### BMDC activation

Cells were isolated from the bone marrow of C57BL/6J mice and were cultured in RPMI 1640 medium supplemented with 20 ng/mL GM-CSF and 10 ng/mL IL-4. The culture medium was replaced and the cytokines were replenished every two days. Nonadherent and loosely adherent cells were harvested on Day 8 and plated in 24-well plates. The cells (1×10^6^ cells/mL/well) were cultured with compounds (0.1% DMSO served as a control) for 24 h at 37 °C and stained with fluorescein-conjugated anti-CD11c-PerCP/Cy5.5 (cat. no. 117328; BioLegend, San Diego, USA), anti-CD40-APC (cat. no. 124612; BioLegend), anti-CD80-FITC (cat. no. 104706; BioLegend) and anti-CD86-BV650 (cat. no. 105036; BioLegend).

### Cell proliferation assay

Mouse spleen lymphocytes (4×10^5^ cells/100 µL/well), BMDCs (2×10^4^ cells/100 µL/well), B16 cells (6×10^3^ cells/100 µL/well), A375 cells (6×10^3^ cells/100 µL/well), DaudiB cells (1.5×10^4^ cells/100 µL/well), A20 cells (1.5×10^4^ cells/100 µL/well) and MCF-10A cells (8×10^3^ cells/100 µL/well) were seeded in 96-well plates. The cells were cultured with various compounds for 48 h at 37 °C (cells treated with DMSO served as a control). Cell Counting Kit-8 reagent (cat. no. MA0218-3; meilunbio, Dalian, China) was then added. The IC_50_ values were calculated by GraphPad.

### Apoptosis and cell cycle assay

B16 cells (5×10^5^ cells/2 mL/well) and A375 cells (5×10^5^ cells/2 mL/well) were treated with compounds (DMSO served as a control) at the indicated concentrations for 24 h. The cells were trypsinized, washed in cold PBS and then pelleted. Some cells were stained with Annexin V-FITC and PI (cat. no. AD10; Dojindo, Kumamoto, Japan). Other samples of the cells were fixed in cold 75% ethanol. After incubation at -20 °C for 4 h, the cells were stained with PI/RNase (cat. no. C543; Dojindo). Both apoptosis and cell cycle progression were determined by an Attune NxT Flow Cytometer (Life Technologies, Waltham, USA).

### RNA sequencing and RT-qPCR

After treatment with compounds (DMSO served as a control) for 24 h, the cells were lysed in TRIzol solution. RNA was extracted using an RNAiso Plus kit (TaKaRa, Kyoto, Japan). Genetic analysis was performed by the BGI-Huada Genomics Institute (Wuhan, China). mRNA enrichment, single-stranded circular DNA library construction, sequencing (DNBSEQ platform) and sequencing data filtering (SOAPnuke software) were performed as previously described 19. Then, quantitative gene analysis and various analyses (principal component analysis, correlation, differentially expressed gene screening, etc.) were performed based on gene expression levels, and KEGG pathway enrichment and classification analysis were performed on the genes that were differentially expressed among the selected samples. cDNA synthesis and real-time PCR were performed using a cDNA synthesis master mix kit and 2×SYBR Green PCR Mix (Simgen, Hangzhou, China). The following primers were used: c-Met: forward, 5′-GGT GCG GTC TCA ATA TCA GTA G-3′, reverse, 5′-CTCTTG CGT CAT AGC GAA CT-3′; β-catenin: forward, 5′-ATGGAGCCGGACAGAAAAGC-3′, reverse, 5′-TGGGAGGTGTCAACATCTTCTT-3′; Mitf: forward, 5′-TAC AGA AAG TAG AGG GAG GAG GAC TAA G-3′, reverse, 5′-CAC AGT TGG AGT TAA GAG TGA GCA TAG CC-3′; and β-actin: forward, 5′-ACTATTGGCAACGAGCGGTT-3′, reverse, 5′-ATGGATGCCACAGGATTCCA-3′ (Sangon Biotech, Shanghai, China). Real-time PCR was carried out using the Step One Plus real-time PCR system (LightCycler 96, Basel, Switzerland).

### Western blotting

After treatment with various compounds (0.2% DMSO served as a control), the cells were lysed with cell lysis buffer. The antibodies used were antibodies against Akt (9272S; CST, Danvers, USA), pAktSer473 (4060S; CST), Met (ab216330; Abcam, Cambridge, UK), β-catenin (8480S; CST), non-phospho (active) β-catenin (Ser45) (19807S; CST), non-phospho (active) β-catenin (Ser33/37/Thr41) (8814S; CST), MiTF (ab140606; Abcam), TRP2/DCT (ab221144; Abcam), and β-actin (9272S; CST), and the target bands were detected with a chemiluminescence system (Clinx Science Instruments, Shanghai, China).

### Endotoxin assay

B16 cells (5×10^5^ cells/2 mL/well) were treated with compounds (DMSO served as a control) at 20 μM for 24 h. The endotoxin levels in the cell culture supernatant were determined using a mouse endostatin ELISA Kit (cat. no. ml037221; mlbio, Shanghai, China). The mean endostatin concentrations of the control, ibrutinib, GY100, Combo, and GY161 groups were 6.6×10^-3^, 6.3×10^-3^, 6.7×10^-3^, 6.8×10^-3^, and 7.2×10^-3^ EU/mL, respectively. All the tested compound solutions (dissolved in DMSO) did not contain endotoxins. In addition, the endotoxin content in the cell culture medium (DMEM+10% FBS) was 8.9×10^-3^ EU/mL. The content of endotoxin was much lower than 5.0 EU/mL (standard level of endotoxin in culture medium: ≤ 5.0 EU/mL).

### *In vivo* antitumor activity

Female C57BL/6J mice (4-6 weeks) were purchased from Medical Laboratory Animal Center (Guangzhou, China). The animal protocols were approved by the Laboratory Animal Ethics Committee of Shenzhen University. B16 cells at a density of 2×10^5^ were implanted subcutaneously into the right flank of each mouse. The mice were randomized into five groups (5-6 mice/group) on day 5 after inoculation. The mice were treated with ibrutinib (10 mg/kg), GY100 (10 mg/kg), Combo (ibrutinib 10 mg/kg and GY100 10 mg/kg) and GY161 (20 mg/kg) via peritumoral injection every other day for a total of five treatments. The control group was treated with an equal volume of solvent (5% DMSO+30% PEG300+65% ddH_2_O). The body weight and tumor size were measured every two days. At the end of the study, the mice were sacrificed. Long-term survival was evaluated until the mice died naturally.

### Detection of CD8^+^ T lymphocytes

At the time of sacrifice, the tumors and spleens were harvested from the mice. Single-cell suspensions of splenocytes were stained with anti-CD3-FITC (cat. no. 11-0032-82; eBioscience, San Diego, USA), anti-CD4-PerCP/Cy5.5 (cat. no. 100540; BioLegend) and anti-CD8a-APC (cat. no. 17-0081-83; eBioscience) at 4 °C for 20 min. The single-cell suspensions of tumors were stained with anti-CD45-R-PE (cat. no. 147711; BioLegend), anti-CD3-FITC, anti-CD8a-APC and anti-IFN-γ-Bv421 (cat. no. 505830; BioLegend) at 4 °C for 20 min. The cells were analyzed by an Attune NxT Flow Cytometer.

### Statistical analysis

Two-way ANOVA was used for differential analysis of the tumor volumes. IC_50_ values were calculated using a nonlinear regression model in GraphPad Prism 5. The data from other experiments were analyzed by unpaired t test, one-way ANOVA with Bonferroni/Dunn post hoc test or log-rank (Mantel-Cox) test. The data are expressed as the mean ± SE. A value of P<0.05 was considered statistically significant.

## Results

### GY161 induced stimulation of TLR7 signaling and cytokine release *in vitro*

**Figure 1:**
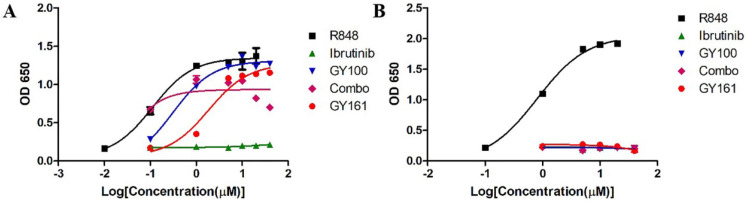
** GY161 stimulated TLR7 signaling. (A)** HEK-Blue hTLR7 cells were treated overnight with the compounds at the indicated concentrations, and the final OD values were recorded at 650 nm. **(B)** Effects of the compounds on HEK reporter cells expressing human TLR8. The data are presented as the mean ± SE (n = 3). Combo: a physical mixture of ibrutinib and GY100 (1:1 molar ratio).

**Figure 2:**
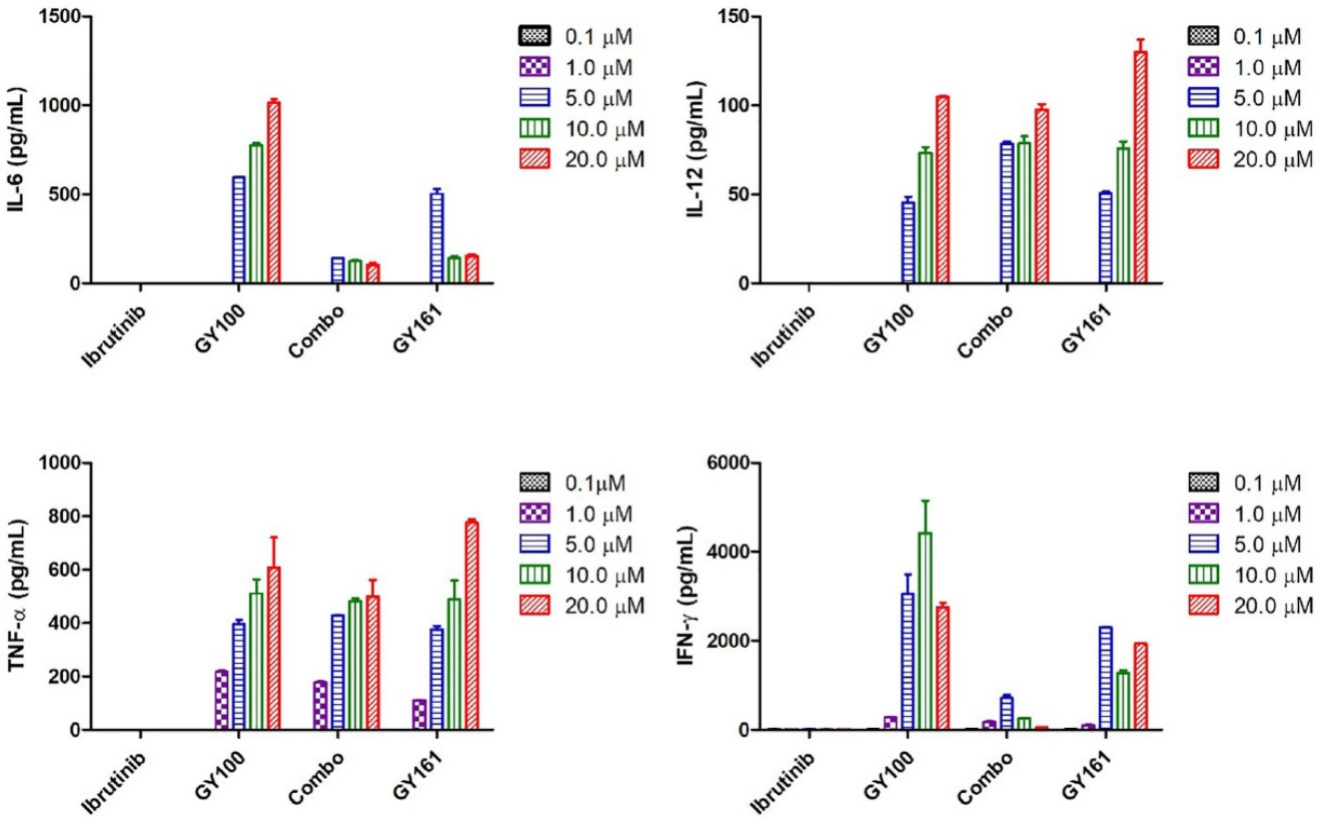
** Mouse spleen lymphocytes were treated with the compounds for 24 h, and IL-6, IL-12, TNF-α and IFN-γ secretion was quantified by ELISA.** The data are presented as the mean ± SE (n = 3). Combo: a physical mixture of ibrutinib with GY100 (1:1 molar ratio).

We first evaluated the effects of the compounds on the TLR7 signaling pathway. We treated TLR7-NF-κB reporter HEK-293 cells with the compounds and observed that the reporter was activated by both GY100 and GY161 in a similar dose-dependent manner, indicating that GY161 could directly stimulate TLR7 signaling. The 50% maximal effective concentration was calculated for each complex. As indicated in Figure 1A, GY100 and GY161 both activated TLR7 with EC_50_ values of 0.31 and 1.86 μM, respectively, while ibrutinib did not stimulate TLR7. Combo exerted weaker effects on the stimulation of the TLR7 pathway than GY161. R848 (resiquimod) is a Toll-like receptor 7 and 8 (TLR7/8) agonist. R848, a positive control, could activate both the TLR7 and TLR8 pathways with EC_50_ values of 0.1 and 0.9 μM, respectively. As expected, TLR8 could not be activated by our compounds, as demonstrated in Figure 1B. Then, the immunological activity of GY161 was evaluated. Mouse spleen-derived lymphocytes were incubated with the compounds for 24 h, and then, the release of cytokines was determined by ELISAs. As shown in Figure 2, GY100 showed a strong effect on the secretion of IL-6, IL-12, TNF-α and IFN-γ. Ibrutinib is poorly immunogenic and rarely stimulates cytokine production. Interestingly, GY161 induced the secretion of nearly the same notably high levels of IL-12 and TNF-α as GY100 in a concentration-dependent manner. Moreover, GY161 induced obviously higher secretion of IL-6 and IFN-γ than Combo at a concentration of 5.0 μM, although this effect was weaker than that of GY100.

### GY161 induced the maturation of BMDCs *in vitro*

**Figure 3:**
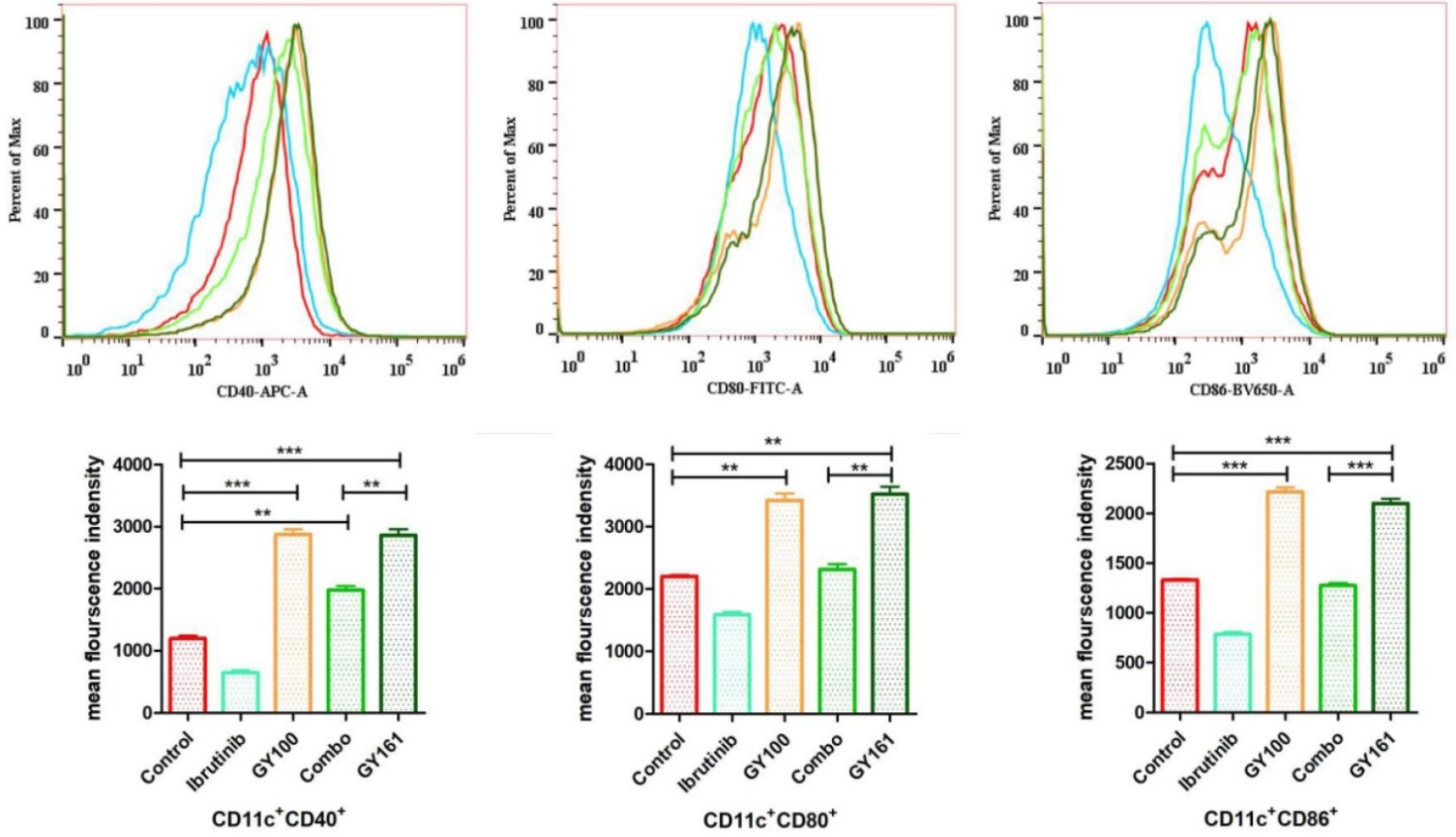
** GY161 could induce BMDC maturation.** Maturation of BMDCs was evaluated by measuring the expression of the surface molecules CD40, CD80 and CD86 by flow cytometry after 24 h of coculture with 0.1% DMSO (control) or compounds (10.0 µM). Significant differences among the groups were analyzed using one-way ANOVA with Bonferroni post hoc test. The data are presented as the mean ± SE, where *P < 0.05, **P< 0.01, ***P<0.001. Combo: a physical mixture of ibrutinib with GY100 (1:1 molar ratio).

Maturation of BMDCs was assessed by flow cytometry by detecting the upregulated expression of the costimulatory molecules CD40, CD80 and CD86, which are necessary for antigen presentation to T cells, after 24 h of coculture with the compounds (10.0 μM). GY161 treatment induced the upregulation of the CD40, CD80 and CD86 levels and showed a stimulatory capacity that was similar to that of GY100. Ibrutinib downregulated the expression of these costimulatory molecules. All the statistical analysis results are shown in Figure 3.

### *In vitro* antiproliferation activity of compound GY161

**Figure 4:**
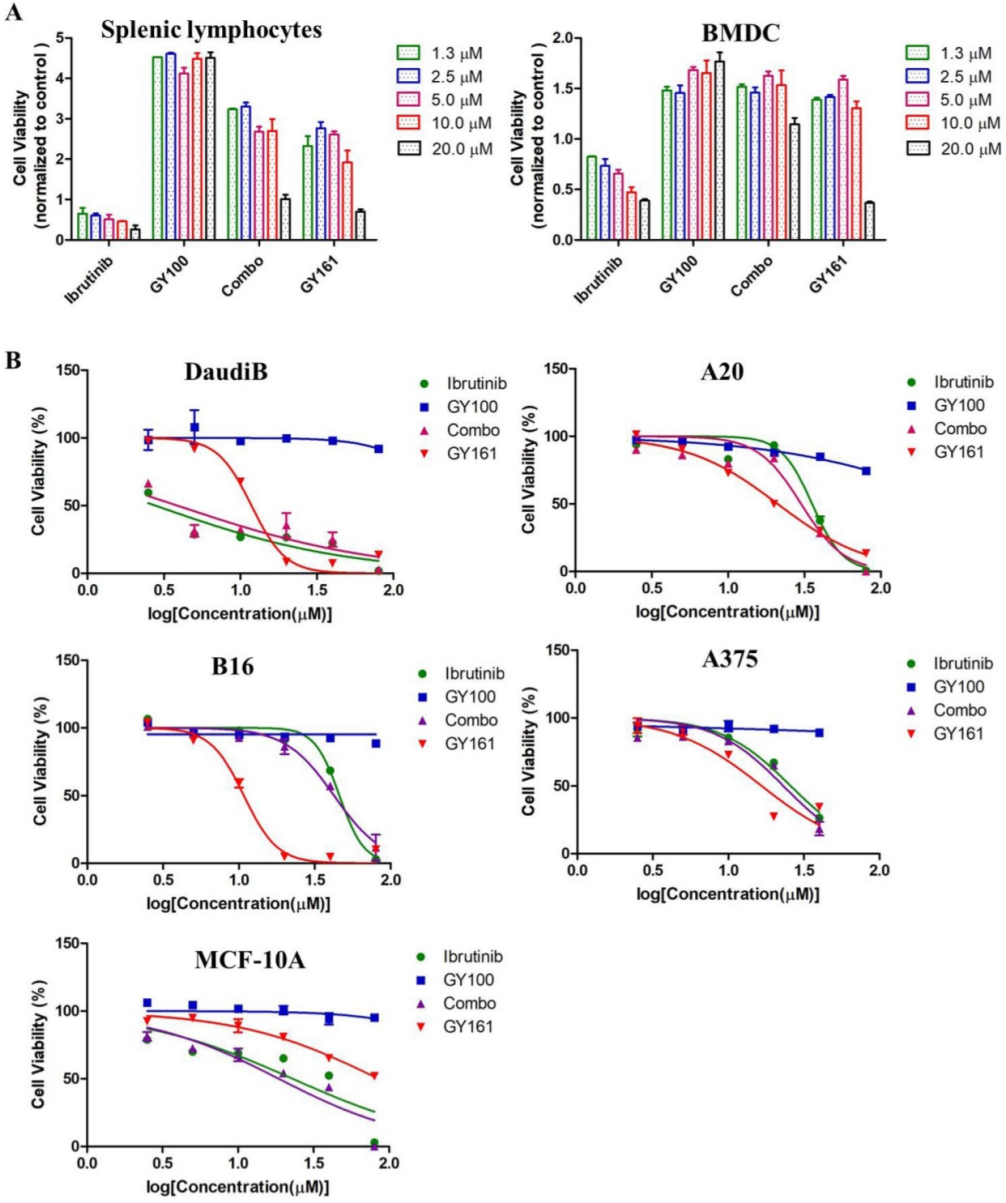
** (A)** Effects of compounds on mouse splenic lymphocyte and BMDC proliferation. Lymphocytes and BMDCs were treated with the compounds for 48 h. **(B)** Antiproliferative effects of selected compounds on DaudiB, A20, B16, A375 and MCF-10A cells. The cells were incubated with serial dilutions of the compounds at various concentrations (2.5, 5.0, 10.0, 20.0, 40.0 and 80.0 µM) for 48 h. The cells were incubated with DMSO as a control. The data are presented as the mean ± SE. Combo: a physical mixture of ibrutinib with GY100 (1:1 molar ratio).

**Figure 5:**
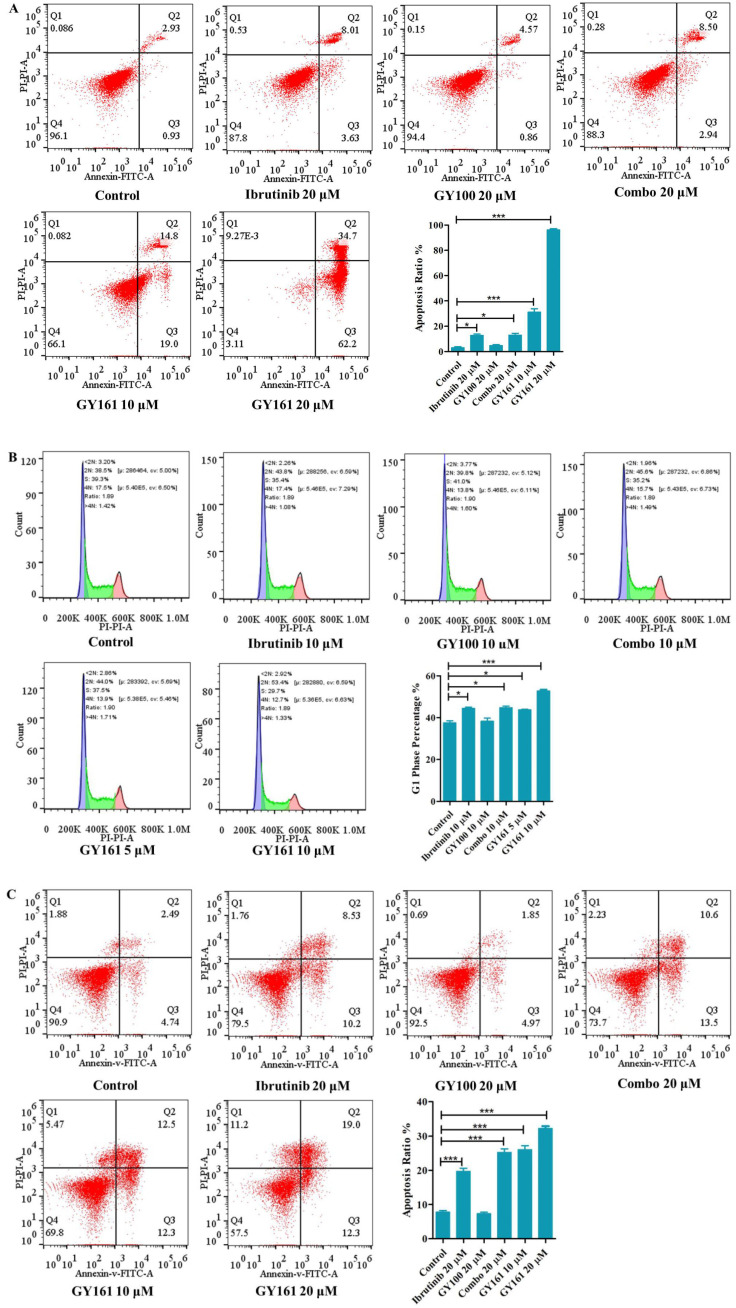

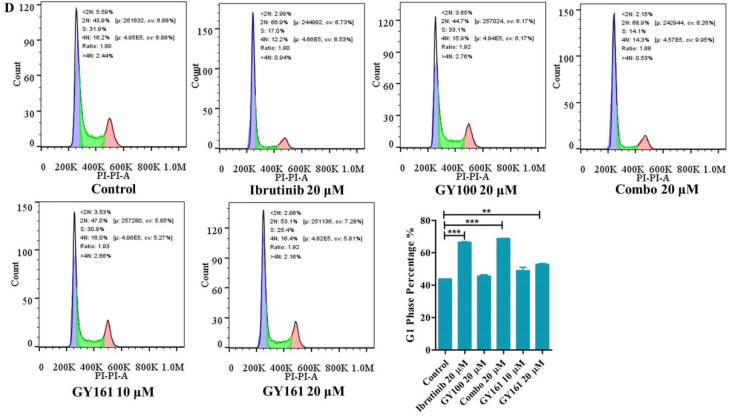
** Effects of compound GY161 on B16 and A375 cells. (A, B)** B16 cells were treated with selected compounds for 24 h, and cell apoptosis and cell cycle progression were analyzed by flow cytometry. **(C, D)** A375 cells were treated with selected compounds for 24 h, and cell apoptosis and cell cycle progression were analyzed by flow cytometry. Cells were treated with DMSO as control. Significant differences among the groups were analyzed using one-way ANOVA with Dunn's post hoc test. The data are presented as the mean ± SE. *P < 0.05, **P< 0.01, ***P<0.001. Combo: a physical mixture of ibrutinib with GY100 (1:1 molar ratio).

As shown in Figure 4A, GY100, Combo and GY161 (at concentrations of from 1.3 to 10.0 μM) substantially promoted the proliferation of splenic lymphocytes and BMDCs, but ibrutinib showed an obvious inhibitory effect on proliferation at these concentrations. The antiproliferative activities of the compounds on B cell lymphoma cells (DaudiB and A20 cells) were first evaluated. The results in Table 1 and Figure 4B indicated that GY161 showed obviously weaker inhibitory activity on DaudiB cells than ibrutinib, and it also did not exert strong antiproliferative effects on A20 cells. We further investigated the activities of the selected compounds against two melanoma cell lines (B16 and A375 cells). GY161 suppressed the proliferation of B16 cells in the micromolar range with an IC_50_ value of 10.79 μM, whereas ibrutinib and Combo showed weaker IC_50_ values of 45.95 μM and 42.38 μM, respectively. Furthermore, the IC_50_ value of GY161 on MCF-10A cells was 85.05 μM, which suggested that GY161 had no obvious toxicity in human normal breast cells. In addition, GY100 did not inhibit any of the cell lines. The effects of GY161 on the apoptosis and cell cycle progression of B16 and A375 cells were then tested (Figure 5A-5D). In B16 cells, GY161 triggered apoptosis in a concentration-dependent manner, with apoptotic ratios of 33.8% and 96.9% at concentrations of 10.0 μM and 20.0 μM, respectively, compared with the apoptotic ratio of 3.9% observed in the control cells. The apoptotic ratio induced by GY161 at a concentration of 20.0 μM was significantly higher than that induced by other treatments. GY161 was also found to significantly arrest cell cycle progression in the G1 phase in a dose-dependent manner. In A375 cells, 20.0 μM GY161 also caused obvious cell apoptosis and cell cycle arrest in the G1 phase, but the percentage of cells in the G1 phase was lower than that after ibrutinib and Combo treatment.

### Compound GY161 inhibited c-Met and its downstream signaling pathway

**Figure 6:**
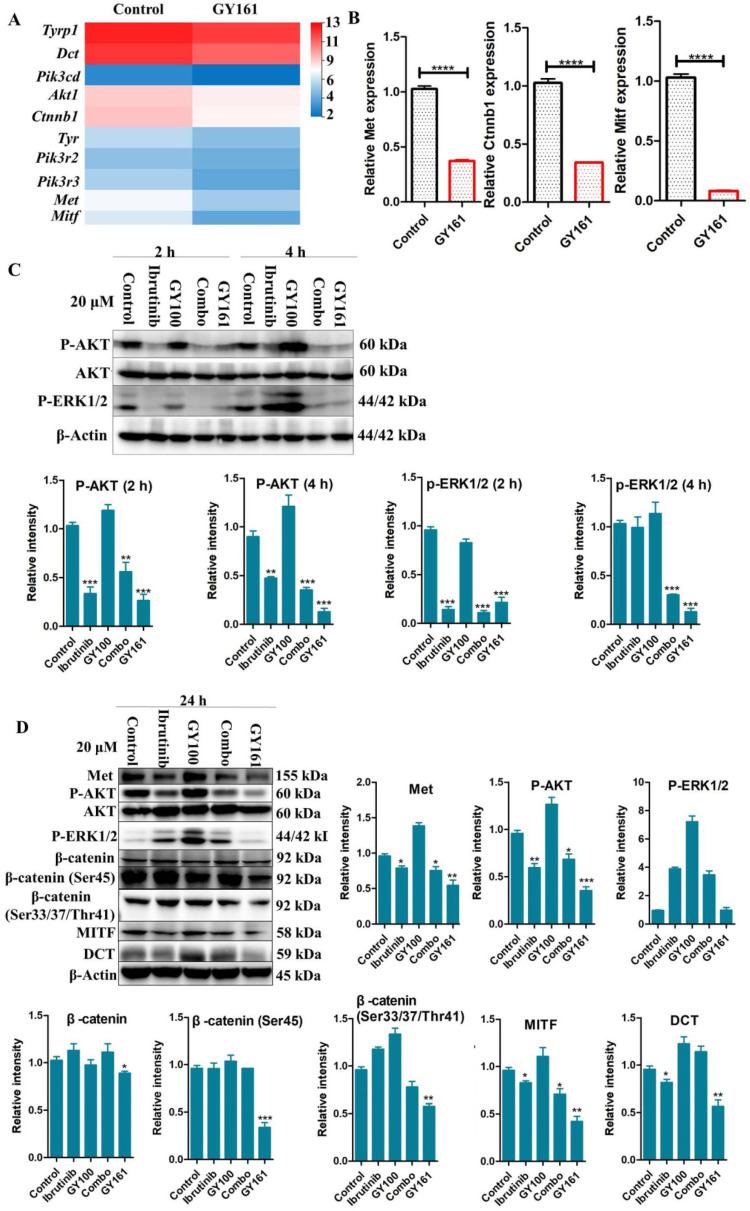
** Effects of the selected compounds (20.0 μM) on c-Met and downstream signaling pathways in B16 cells. (A)** A heatmap of the expression of ten genes in the control and GY161 groups. **(B)** The relative expression levels of Met, Ctnnb1 and Mitf were detected by qPCR. **(C, D)** c-Met protein expression and the inhibition of c-Met/β-catenin signaling in GY161-treated B16 cells were assessed by western blotting. Cells were treated with DMSO as control. Significant differences among the groups were analyzed using a T test (for B) and one-way ANOVA with Dunn's post hoc test (for C and D). The data are presented as the mean ± SE. *P< 0.05, **P< 0.01, ***P<0.001, ****P<0.0001 versus control. Combo: a physical mixture of ibrutinib with GY100 (1:1 molar ratio).

RNA sequencing was performed in B16 cells to further examine the effects of the compounds on cell signaling. First, differentially expressed genes between the GY161 group and the control group were identified. Using KEGG pathway classification to analyze these differentially expressed genes, we found that the number of genes involved in signal transduction was the highest (700 genes). Then, the top 20 most variable pathways in signal transduction were confirmed via KEGG enrichment analysis (Supplemental Figure S3). After careful analysis of these pathways, we discovered that the expression of many genes enriched in the PI3K-Akt, Ras-MAPK and Wnt pathways was changed markedly after treatment with GY161 (Supplemental Figure S4). Specifically, we observed that the expression of Met, Pik3cd, Pik3r3, Pik3r2, Akt1, Ctnnb1, Hras, Nras and Mapk1 was decreased significantly by GY161 (Figure 6A and Supplemental Table S1). In addition, it was found that the expression of genes (Mitf, Tyr, Tyrp1 and Dct) in the melanogenesis pathway, which is associated with the MAPK and Wnt pathways, was notably downregulated in the GY161 group. The GY161-induced downregulation of Met, Ctnnb1 and Mitf expressed in B16 cells was confirmed by both qPCR (Figure 6B) and western blotting (Figure 6C and 6D). We found that GY161 at a concentration of 20.0 μM significantly decreased the protein level of c-Met. Moreover, similar effects on phosphorylated Akt and ERK1/2 were observed for GY161, as expected. ERK1/2 and AKT are key downstream molecules of c-Met that play important roles in c-Met function. Notably, ibrutinib and GY100 resulted in complex changes in ERK1/2 phosphorylation; they exerted inhibitory effects at 2 h and exerted activating effects over time. Next, we examined whether GY161 affects the protein levels of β-catenin. The western blotting results showed that GY161 significantly decreased the protein levels of β-catenin (active) and its downstream molecules MITF and DCT (Figure 6D). These data indicated that GY161 inhibited c-Met activity as well as subsequent c-Met downstream signaling.

### *In vivo* antitumor activity of compound GY161

**Figure 7:**
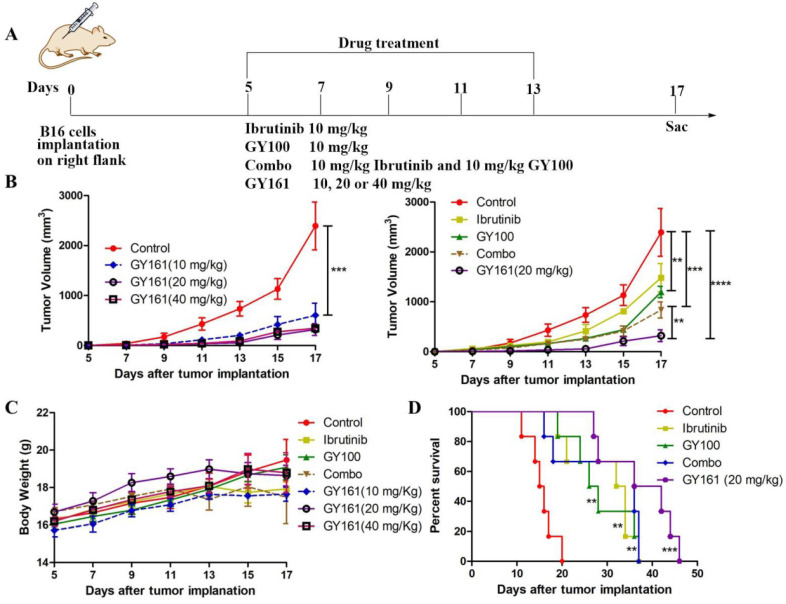
** GY161 reduces tumor burden in B16 tumor-bearing mice. (A)** Treatment scheme. Six- to 8-week-old C57BL/6J mice were inoculated with 2 × 10^5^ B16 cells. **(B)** Tumor growth curves were measured with a digital caliper (n = 5 mice per group). **(C)** The body weights of the group mice over time. **(D)** Kaplan-Meier survival plots of the treated mice (n = 6 mice per group). GY161 prolonged the survival time of mice. The control group was treated with an equal volume of solvent (5% DMSO+30% PEG300+65% ddH_2_O). Significant differencesces among the groups were analyzed using two-way ANOVA with Bonferroni post hoc test (for B) and log-rank (Mantel-Cox) test (for D). **P< 0.01, ***P<0.001, ****P<0.0001.

Based on the potent antiproliferative efficacy of the compound *in vitro*, the *in vivo* antitumor activity of GY161 was further investigated in mice with B16 melanoma. As shown in Figure 7B, the control, ibrutinib, GY100, Combo, 10 mg/kg GY161, 20 mg/kg GY161 and 40 mg/kg GY161 groups developed mean final tumor volumes of 2392.57, 1483.88, 1198.27, 835.86, 604.99, 320.74 and 346.99 mm^3^, respectively, at the end of 17 days. Monotherapy with ibrutinib or GY100 delayed primary tumor growth. As expected, combination treatment led to a significantly reduced tumor burden compared to monotherapies. Notably, the administration of GY161 achieved the best therapeutic effect compared with all other treatments. The results showed that the 20 mg/kg and 40 mg/kg GY161 groups had no significant changes in tumor volume, and the tumors in these groups were smaller than those in the 10 mg/kg group, at 17 days. In all five groups, the weight of the mice did not decrease significantly during the course of treatment (Figure 7C). Moreover, an obvious increase in mouse survival was observed after treatment with GY161 at a dose of 20 mg/kg compared with the negative control (Figure 7D). Moreover, we found that either therapy alone or the Combo treatment prolonged the survival rate of mice.

### GY161 induced tumor-specific CD8^+^ T cell generation

**Figure 8:**
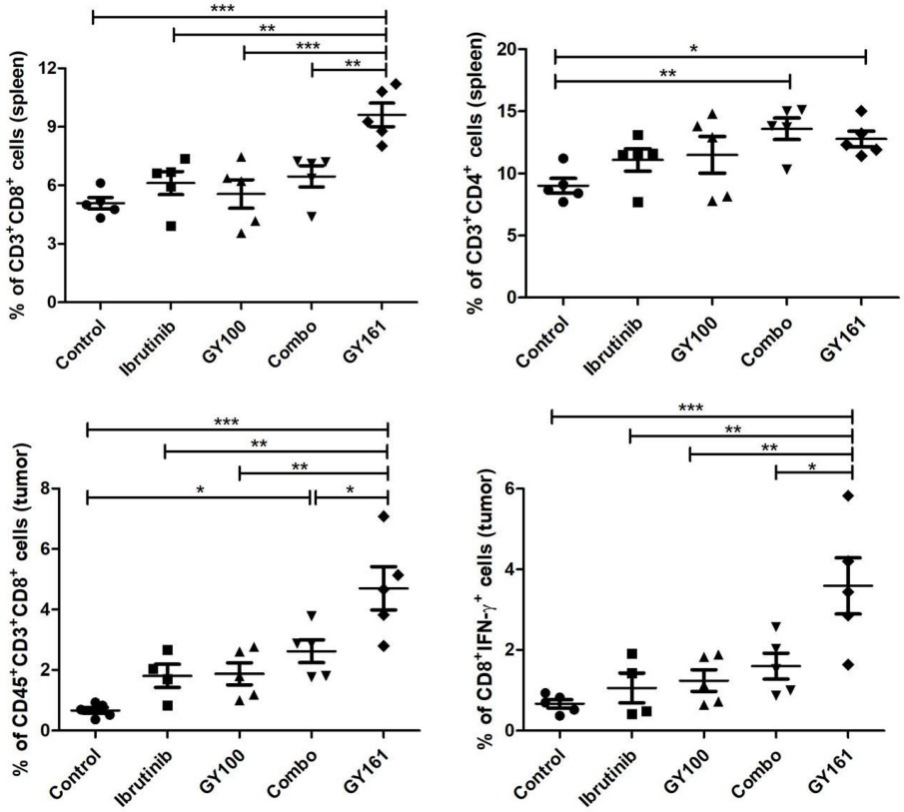
** The effect of GY161 (20 mg/kg) on T lymphocyte numbers in spleens and tumors.** Mice were treated as shown in Figure 7A. Spleens and tumors were harvested on day 17, and T cells were analyzed by flow cytometry. Numbers of CD8^+^ and CD8^+^IFN-γ^+^ T were calculated. Significant differencesces among the groups were analyzed using one-way ANOVA with Bonferroni post hoc test. The data are presented as mean ± SE; *P<0.05, **P<0.01, ***P<0.001.

To investigate the effects of each treatment on cellular immunity of the mice, we determined the numbers of CD8^+^ T cells in the spleens and tumors by flow cytometry. As shown in Figure 8, the percentage of CD8^+^ T cells in the spleens was markedly elevated in the GY161 group compared to the other groups. Furthermore, the Combo group exhibited an additive increase in CD4^+^ T cell numbers compared with the ibrutinib or GY100 groups. We also confirmed that Combo increased CD8^+^ T cell numbers in tumors more than any singular treatment, but notably, GY161 promoted more significant increases in CD8^+^ T cell numbers than all the other treatments. Moreover, compared to the other treatments, GY161 induced a strong increase in CD8^+^IFN-γ^+^ T cell numbers in tumors (Figure 8).

## Discussion

We hypothesized that coupling tumor-targeting drugs with TLR7 agonists could attract and lead immune cells to the tumor microenvironment, specifically stimulate local immunity and simultaneously kill cancer cells. Ibrutinib not only targets cancer-associated kinases but also influences the tumor microenvironment by modulating MDSCs and TAMs 2-7. TLR7 agonists can also regulate these immunosuppressive cells and increase the proportion of infiltrating CD8^+^ T lymphocytes to enhance cancer immunotherapy 20, 21. Moreover, systemic and intratumoral TLR7 treatment is an effective therapy for TLR7-negative tumors, such as those derived from 4T1, B16, and LLC cells 16. Based on the above hypothesis and information, a series of conjugates of ibrutinib with GY100 were synthesized, and their antitumor activity was evaluated *in vitro* (Supplemental Figure S1 and S2). Due to the strong antiproliferative activity of GY161 against B16 cells, we conducted a systematic study of this compound.

In this study, we first demonstrated that 0.1 to 40.0 μM GY161 could activate TLR7-NF-kB signaling in a TLR7-specific system, which suggested that GY161 preserved the ability of GY100 to stimulate TLR7 *in vitro*. It was reported that TLR7 activation could lead to strong Th1-biased immune responses and induce the release of proinflammatory cytokines 22. Consistent with previous research, we also observed that the production of Th1-biased cytokines TNF-α, IFN-γ and IL-12 and one Th2-biased cytokine, IL-6, by splenic lymphocytes was significantly induced upon treatment with GY161. The significant secretion of proinflammatory cytokines promotes the proliferation and activity of cytotoxic T cells. DCs are professional antigen-presenting cells that play a crucial role in shaping the adaptive immune response 23. In the presence of costimulatory molecules, antigen presentation by DCs results in the activation of tumor antigen-specific cytotoxic T lymphocytes (CTLs) 24. Importantly, GY161 showed a comparable stimulatory capacity compared to GY100.

Cytotoxicity was also assessed in immune cells and tumor cells, as shown in Figure 4. GY161 was not only nontoxic to splenic lymphocytes and BMDCs but also promoted their proliferation at concentrations ranging from 1.3 to 10.0 μM. However, ibrutinib showed an obvious proliferation inhibitory effect at these concentrations. By screening GY161 in four cancer cell lines, we unexpectedly found that GY161 resulted in stronger inhibition of B16 cell proliferation than ibrutinib and Combo. GY161 did not exert a stronger growth inhibitory effect on B cell lymphomas than ibrutinib. We also proved that GY161 had no obvious toxicity in human normal breast cells. Moreover, GY161 was found to significantly trigger in B16 cell apoptosis and cell cycle arrest in the G1 phase in a concentration-dependent manner. These findings prompted us to further explore the mechanisms by which GY161 affects B16 cells.

**Figure 9:**
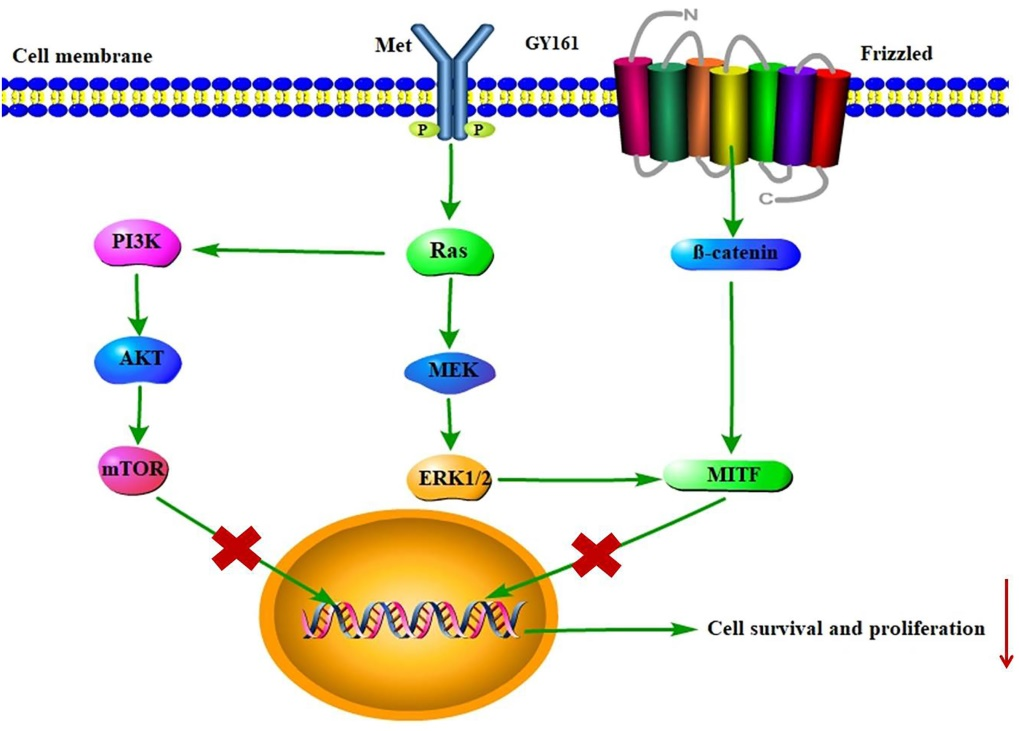
Proposed mechanism by which GY161 regulates B16 cells *in vitro*. We proposed that GY161 inhibited tumor growth by regulating the c-Met/β-catenin pathway, which is associated with B16 cell proliferation and survival.

RNA sequencing was used to further elucidate the mechanisms underlying the antitumor effects of GY161. After GY161 treatment, the gene expression levels of Met, Pik3cd, Pik3r3, Pik3r3, Akt1, Ctnnb1, Mitf, Tyr, Tyrp1 and Tyrp2 in B16 cells were decreased significantly. We further verified the levels of the proteins these genes encode. As shown in Figure 6, GY161 effectively inhibited the signaling pathways downstream of c-Met, since the expression levels of c-Met, p-AKT and p-ERK decreased. Significantly decreased protein levels of β-catenin (active) and its downstream molecules, MITF and DCT, were also observed 25. Ibrutinib had no effect on the expression of β-catenin (active) and a complex effect on ERK1/2 proteins, which may be the reason for the reduced activity of ibrutinib on B16 cells compared with the activity of GY161. It has been demonstrated that activation of the HGF/c-Met, WNT/β-catenin, MAPK/ERK and PI3K/AKT pathways is crucial for melanoma development 26, 27. Moreover, the crosstalk between these pathways makes the identification of therapeutic targets more complicated 28. Encouragingly, our results demonstrated that GY161 exerted inhibitory effects on key proteins in these pathways, especially on the well-known oncogene Met (Figure 9).

The *in vivo* antitumor activity of compound GY161 was evaluated in the B16 xenograft model. Peritumoral injection was chosen in our study to achieve a more effective and safer cancer treatment. The *in vivo* evaluation showed that GY161 exhibited notable antitumor efficacies and improved survival relative to either therapy alone or Combo. The cellular immune response mediated by T cells plays a central role in the generation and regulation of the immune response to tumor-associated antigens. CD8^+^ cytotoxic T lymphocytes (CTLs) are considered to be the preferred immune cells for targeting cancer 29. We demonstrated that local GY161 treatment increased cytotoxic tumor-specific CD8^+^ T cell numbers, ultimately inhibiting tumor growth.

## Conclusion

In summary, we rationally designed and carried out innovative work to generate immunogenic small molecule drugs with bifunctional immune stimulating and antitumor targeting abilities. Future studies should extensively screen a series of potential coupling compounds and focus on the relevant chemical approaches, structure-activity relationships and mechanisms underlying intracellular effects.

## Supplementary Material

Supplementary figures and table.

## Figures and Tables

**Table 1:** Antiproliferative activities of compounds against different tumor cell lines and normal cell line

Compounds	IC_50_ values^ a^ (μM)				
	DaudiB	A20	B16	A375	MCF-10A
Ibrutinib	2.78 ± 1.31	35.51 ± 1.06	45.95 ± 1.04	25.85 ± 1.07	22.98 ± 1.32
GY100	>80.00	>80.00	>80.00	>80.00	>80.00
Combo	3.88 ± 1.38	30.09 ± 1.16	42.38 ± 1.06	23.09 ± 1.11	19.13 ± 1.12
GY161	11.82 ± 1.05	21.10 ± 1.06	10.79 ± 1.04	16.60 ± 1.15	85.05 ± 1.09

^a^IC_50_ values were calculated using a nonlinear regression model (log[inhibitor] *vs.* normalized response-variable slope) in GraphPad Prism 5. The data are presented as the mean ± SE (n=3).

## Abbreviations

KEGG: kyoto encyclopedia of genes and genomes
c-Met: c-mesenchymal-epithelial transition factor
AKT: protein kinase B
ERK: extracellular regulated protein kinases
MITF: microphthalmia-associated transcription factor
mTOR: mammalian target of rapamycin
PI3K: phosphatidylinositol-3-kinase
RAS: Rat sarcoma
IC_50_: the half maximal inhibitory concentration
HPLC: high performance liquid chromatography
NMR: nuclear magnetic resonance
