# Supplementary material for: Conjugate of ibrutinib with a TLR7 agonist suppresses melanoma progression and enhances antitumor immunity
Source: Int J Biol Sci. 2022 Jan 1;18(1):166–79. doi: 10.7150/ijbs.64094 (PMC8692160; doi:10.7150/ijbs.64094)
Supplement: Supplementary file 1 — Supplementary figures and table. [file ijbsv18p0166s1.pdf]

Supplementary materials

Conjugate of ibrutinib with a TLR7 agonist suppresses melanoma progression and enhances antitumor immunity

Sumei Ren[1,2], Xiaodong Wang[1]*, Guangyi Jin[1]*

*Corresponding author: Guangyi Jin email: gyjin@szu.edu.cn; Xiaodong Wang email: wangxiaodong@szu.edu.cn

Contents:

1. Chemistry .... …2-4

2. Spectra for all target compounds... ...5-10

3. Antiproliferative effects..11

4. RNA sequencing...11-12

1. Chemistry

To a solution of commercially available A1 (1.0 g, 4.21 mmol) and 3-bromo-1-propyne (600.7 mg, 5.05 mmol) in 5 mL DMF was added K_2CO_3 (1.7 g, 12.63 mmol). Then, the reaction mixture was stirred for 4 h at room temperature and diluted with 10 ml of water, and the precipitate was separated to obtain crude A2. The crude product was dissolved in 2 ml of concentrated hydrochloric acid, stirred for 2 h and then treated with NaOH to adjust the *p*H to 4, precipitating compound GY100 as a white solid (770.0 mg, 70.0% over two steps). To a solution of GY100 (52.4 mg, 0.20 mmol) and commercially available B1 (68.09 mg, 0.20 mmol) in 2.0 mL DMSO/H_2O (4:1, v/v), L-ascorbic acid sodium salt (3.96 mg, 0.02 mol) and $CuSO_4$ (3.19 mg, 0.02 mol) were added. Then, the reaction mixture was stirred for 2 h at room temperature. Crude B2 was subjected to the next step without further purification and was pure enough for structural characterization. DCM (1.0 mL) and trifluoroacetic acid (0.5 mL) were added, and the reaction mixture was stirred for another 1 h at room temperature. The reaction mixture was concentrated *in vacuo* to yield crude B3. Crude B3 was subjected to the next step without further purification. To a solution of B3 in dry DMSO (1.0 mL) were added 1,1'-thiocarbonyldiimidazole (53.5 mg, 0.30 mmol) and triethylamine (55.6 μL, 0.40 mmol). The mixture was stirred at room temperature for 16 h. Commercially available B5 (77.3 mg, 0.20 mmol) was then added, and the solution was stirred for 4 h at room temperature. The reaction mixture was purified by HPLC to yield GY161 as a white solid (55.4 mg, 29.8% over four steps). **GY100:** ^{1}H NMR (600 MHz, DMSO-d_6) δ 10.02 (s, 1H, OH),

6.49 (s, 2H, NH_2), 4.44 (d, *J* = 2.4 Hz, 2H), 4.16 (t, *J* = 6.6 Hz, 2H), 3.22 (t, *J* = 2.4 Hz, 1H), 1.67–1.62 (m, 2H), 1.43–1.37 (m, 2H), 0.94 (t, *J* = 7.2 Hz, 3H, CH_3); ^{13}C NMR (151 MHz, DMSO-d_6) δ 160.08 , 151.52 , 148.49, 147.82, 98.29, 78.63, 73.82, 65.88, 30.57, 28.34, 18.73, 13.70. ESI-MS (*m/z*): 262.1 $[M+H]^+$ (Calcd 262.3).

B2: ^1H NMR (600 MHz, CD_3OD) δ 7.96 (s, 1H), 5.10 (s, 2H), 4.41 (t, *J* = 6.6 Hz, 2H), 4.26 (t, *J* = 6.3 Hz, 2H), 3.09 (t, *J* = 6.5 Hz, 2H), 2.70–2.54 (m, 12H), 1.93–1.90 (m, 2H), 1.73–1.69 (m, 4H), 1.52–1.43 (m, 14H), 0.96 (t, *J* = 7.4 Hz, 3H); ^{13}C NMR (151 MHz, DMSO-d_6) δ 159.95, 155.49, 151.86, 148.89, 147.65, 142.61, 122.96, 98.22, 77.37, 65.70, 56.07, 54.59, 51.42, 48.94, 39.95, 37.88, 34.50, 30.51, 28.15 ($OC(\underline{C}H_3)_3$), 27.33, 25.85, 22.30, 18.66, 13.62.

B4: ^1H NMR (600 MHz, DMSO-d_6) δ 10.09 (d, *J* = 5.8 Hz, 1H), 7.95 (s, 1H), 6.51 (s, 2H), 4.91 (s, 2H), 4.31(t, *J* = 6.6 Hz, 1H, 2H), 4.13 (t, *J* = 6.6 Hz, 1H, 2H), 3.493.34 (m, 4H), 3.08 (q, *J* = 6.6 Hz, 1H), 2.39–2.30 (m, 8H), 1.79–1.74 (m, 2H), 1.64–1.60 (m, 3H), 1.56 (dt, *J* = 14.0, 7.0 Hz, 1H), 1.41–1.32 (m, 4H), 0.91 (td, *J* = 7.3, 1.4 Hz, 3H); ^{13}C NMR (151 MHz, DMSO-d_6) δ 163.40, 161.00, 160.07, 152.00, 149.02, 147.75, 142.68, 123.07, 98.33, 65.82, 56.58, 55.03, 52.26, 52.21, 52.15, 49.14, 35.36, 34.61, 30.62, 27.57, 26.02, 22.73, 18.78, 13.73; ESI-MS (*m/z*): 544.1 $[M+H]^+$ (Calcd 544.7).

GY161: ^1H NMR (600 MHz, CD_3OD) δ 8.24 (s, 1H), 7.93 (s, 1H), 7.69–7.65 (m, 2H, Ph-H), 7.41–7.38 (m, 2H, Ph-H), 7.18–7.12 (m, 3H, Ph-H), 7.08 (dd, *J* = 8.6, 0.9 Hz, 2H, Ph-H), 5.07 (s, 2H), 4.84–4.79 (m, 2H), 4.57 (d, *J* = 13.8 Hz, 1H), 4.39 (t, *J* = 6.6 Hz, 2H), 4.24 (t, *J* = 6.6 Hz, 2H), 3.82 (dd, *J* = 12.8, 10.0 Hz, 1H), 3.71 (t, *J* = 6.7 Hz,

2H), 3.27 (s, 1H), 2.96–2.80 (dd, J = 51.6, 45.1 Hz, 10H), 2.66 (s, 1H), 2.55 (t, J = 7.2 Hz, 2H), 2.37–2.31 (m, 1H), 2.22–2.14 (m, 1H), 2.01–1.98 (m, 1H), 1.94–1.88 (m, 4H), 1.78–1.74 (m, 1H), 1.72–1.67 (m, 2H), 1.51–1.42 (m, 4H), 0.95 (t, J = 7.8 Hz, 3H); ^{13}C NMR (151 MHz, DMSO-d_6) δ 181.00, 159.93, 158.06, 157.01, 156.18, 155.57, 153.83, 151.91, 148.92, 147.59, 143.23, 142.54, 130.02, 129.96, 127.76, 123.68, 122.93, 118.88, 118.84, 98.19, 97.25, 65.70, 56.27, 55.45, 51.95, 51.82, 51.67, 51.21, 48.98, 47.16, 44.32, 40.31, 39.93, 34.50, 30.49, 29.79, 27.38, 24.79, 23.53, 22.55, 18.65, 13.61; ESI-MS (m/z): 931.3 [M+H]$^+$ (Calcd 931.1).

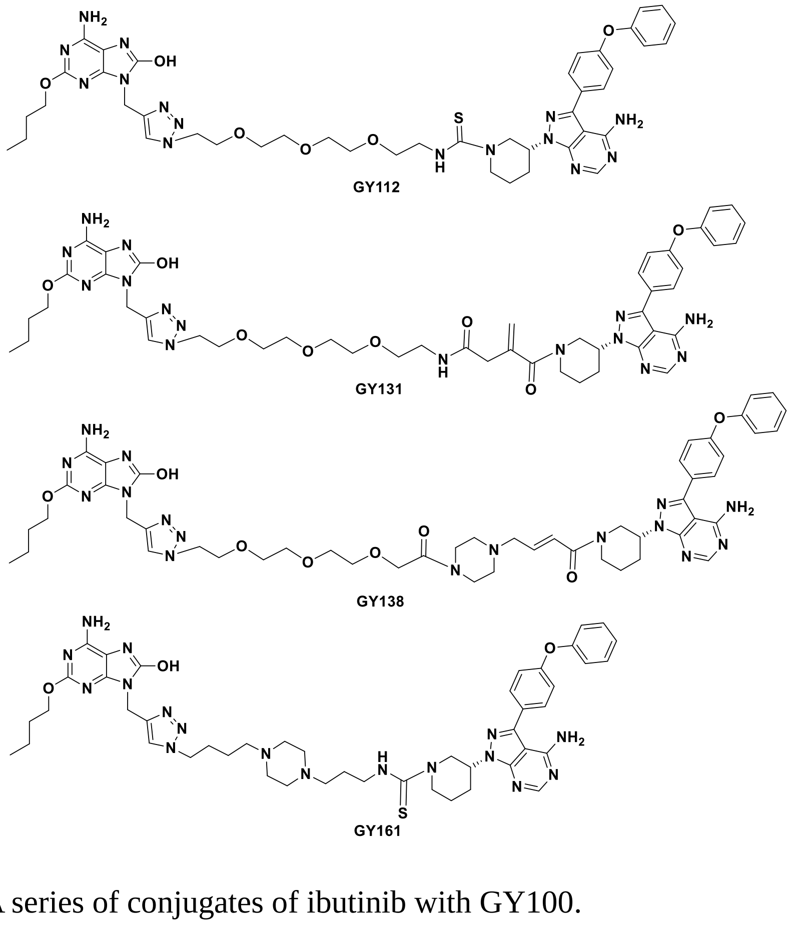

Figure S1. A series of conjugates of ibutinib with GY100.

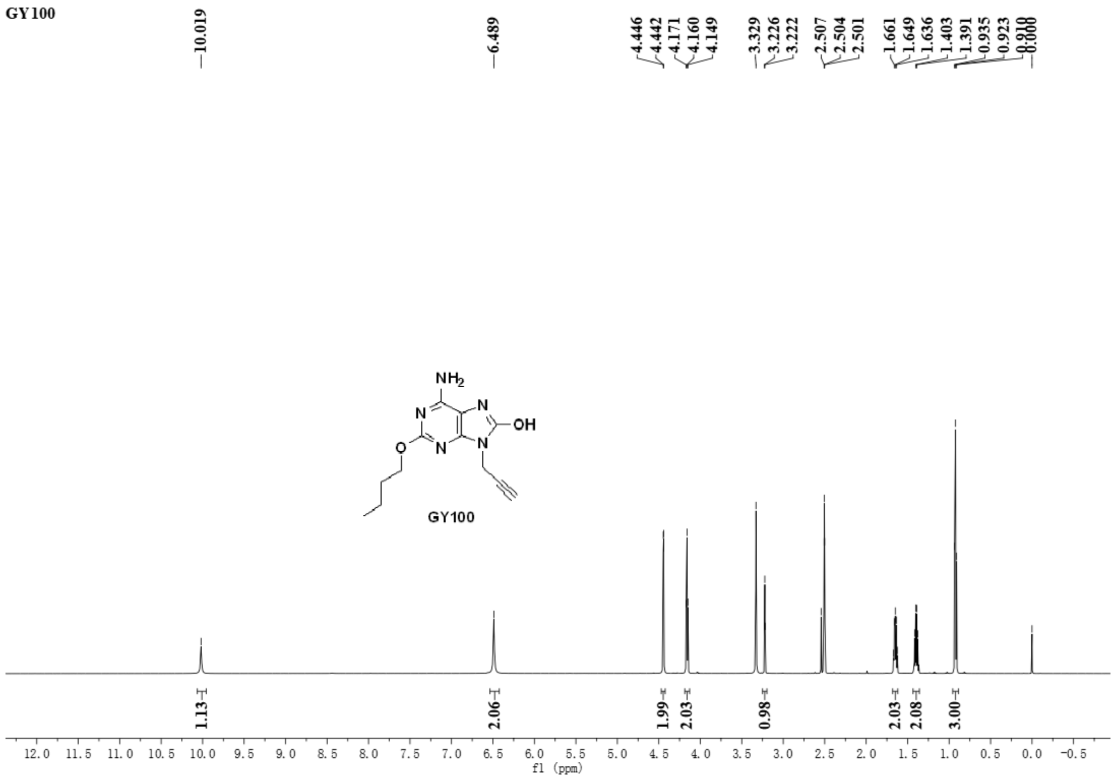

[1] H NMR of **GY100** (600 MHz, DMSO-d_6)

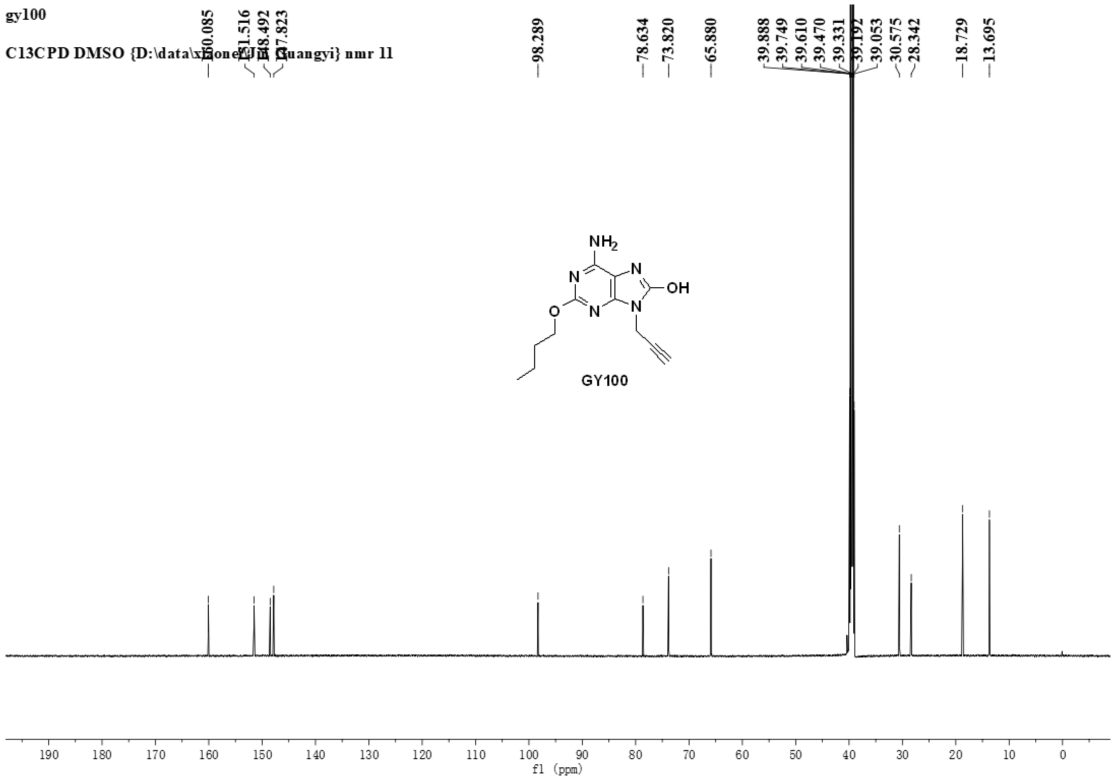

[13] C NMR of **GY100** (151 MHz, DMSO-d_6)

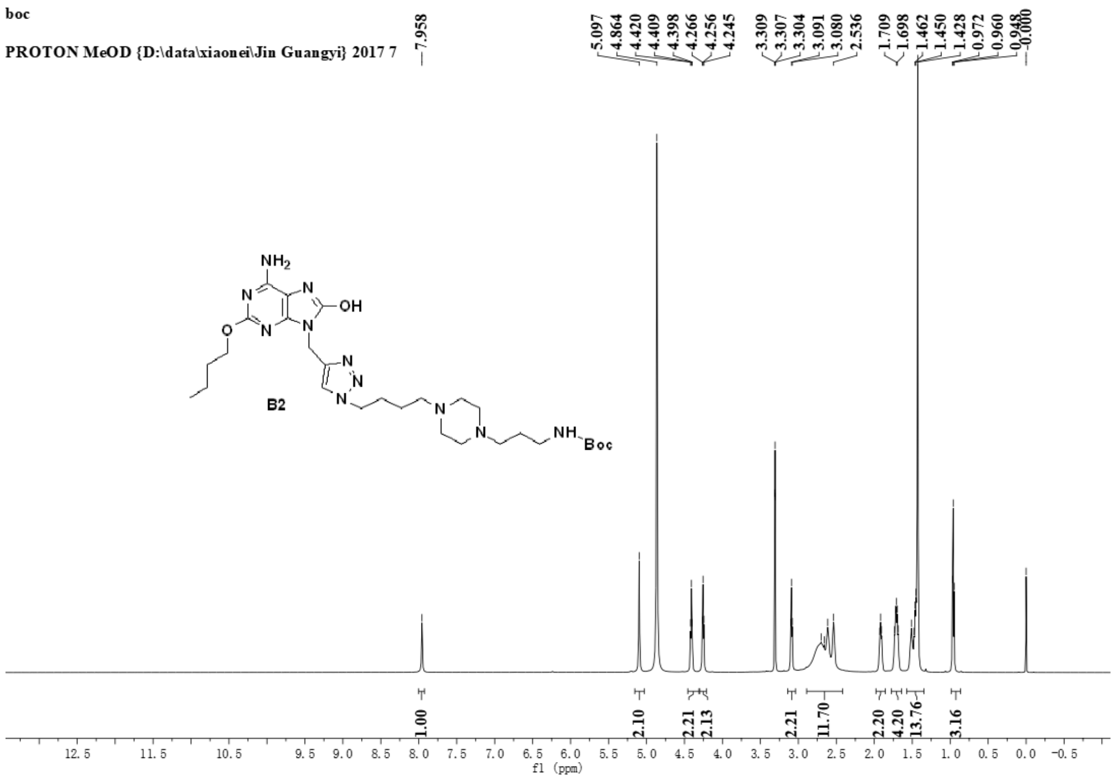

[1] H NMR of **B2** (600 MHz, CD_3OD)

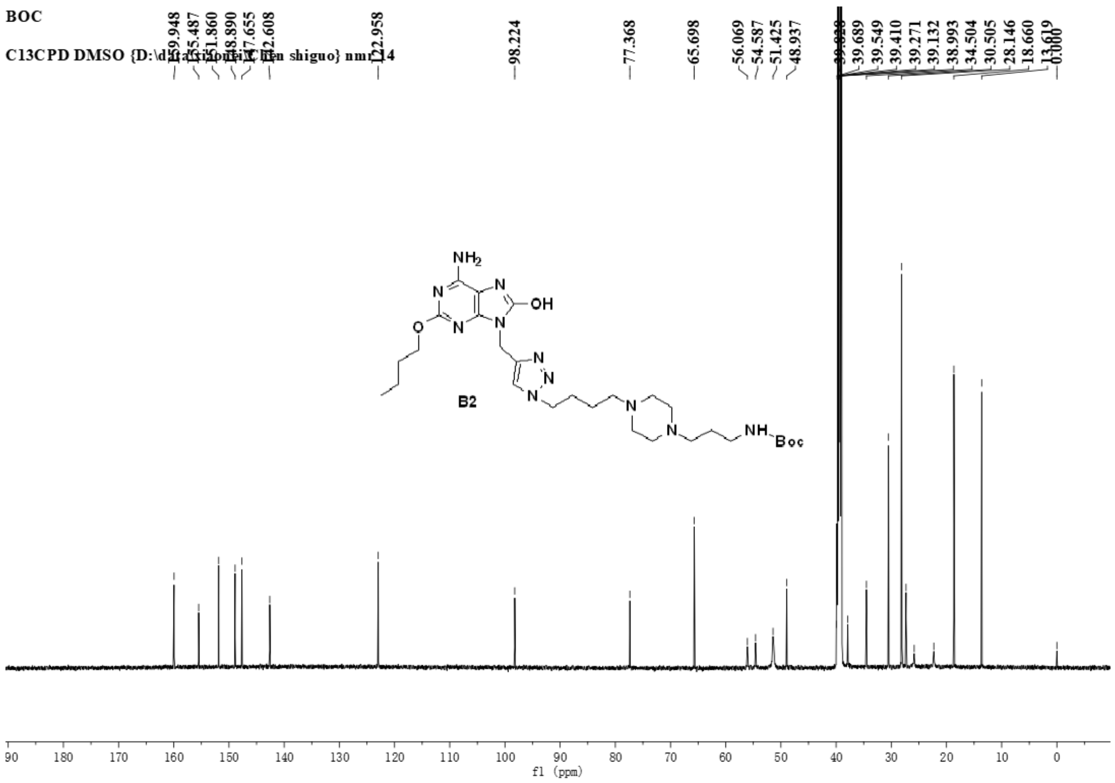

[13] C NMR of **B2** (151 MHz, DMSO-d_6)

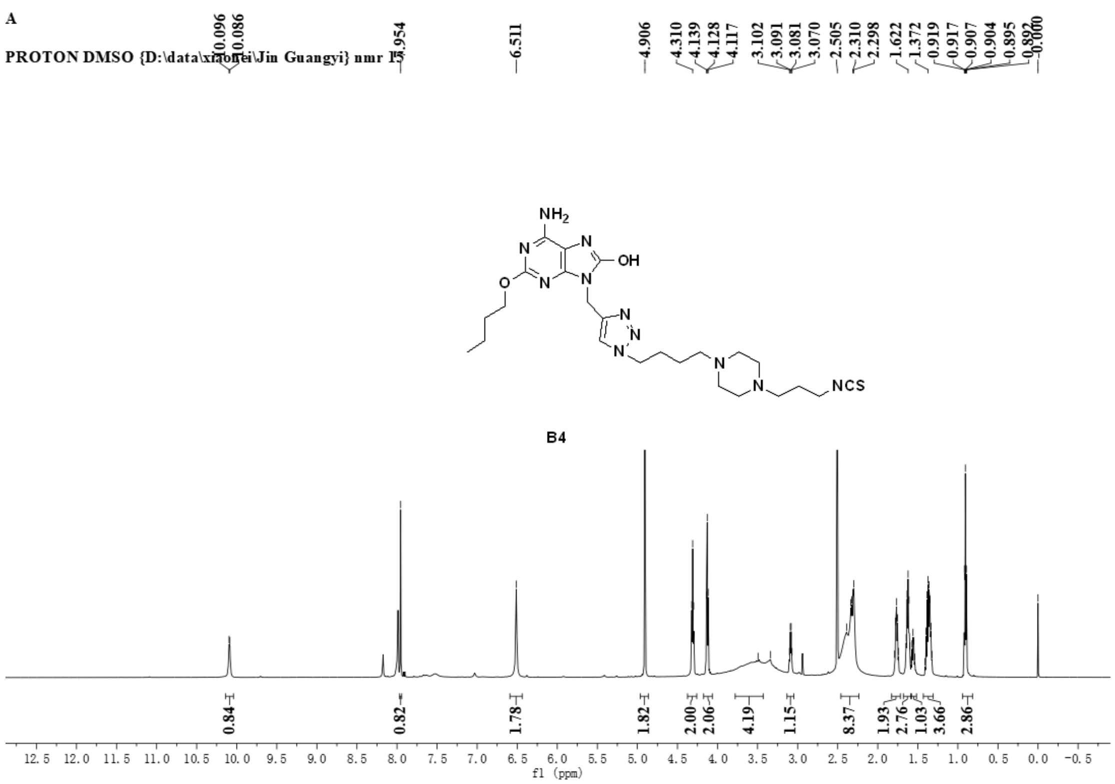

[1] H NMR of **B4** (600 MHz, DMSO-d_6)

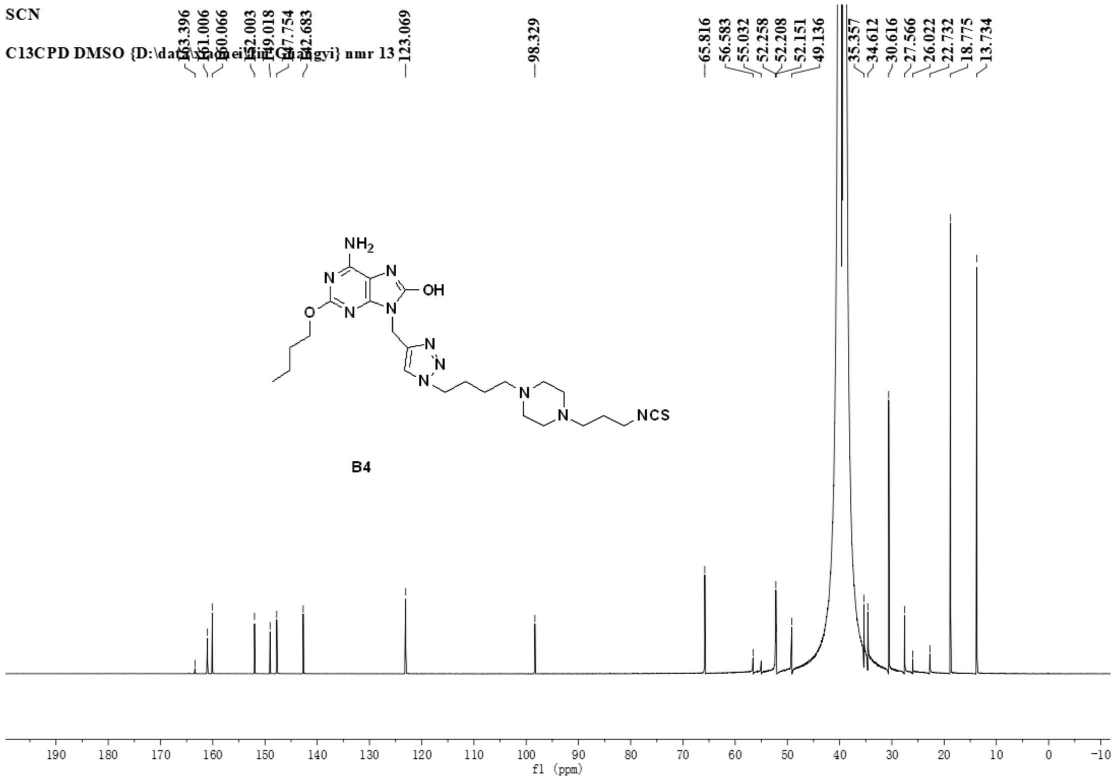

[13] C NMR of **B4** (151 MHz, DMSO-d_6)

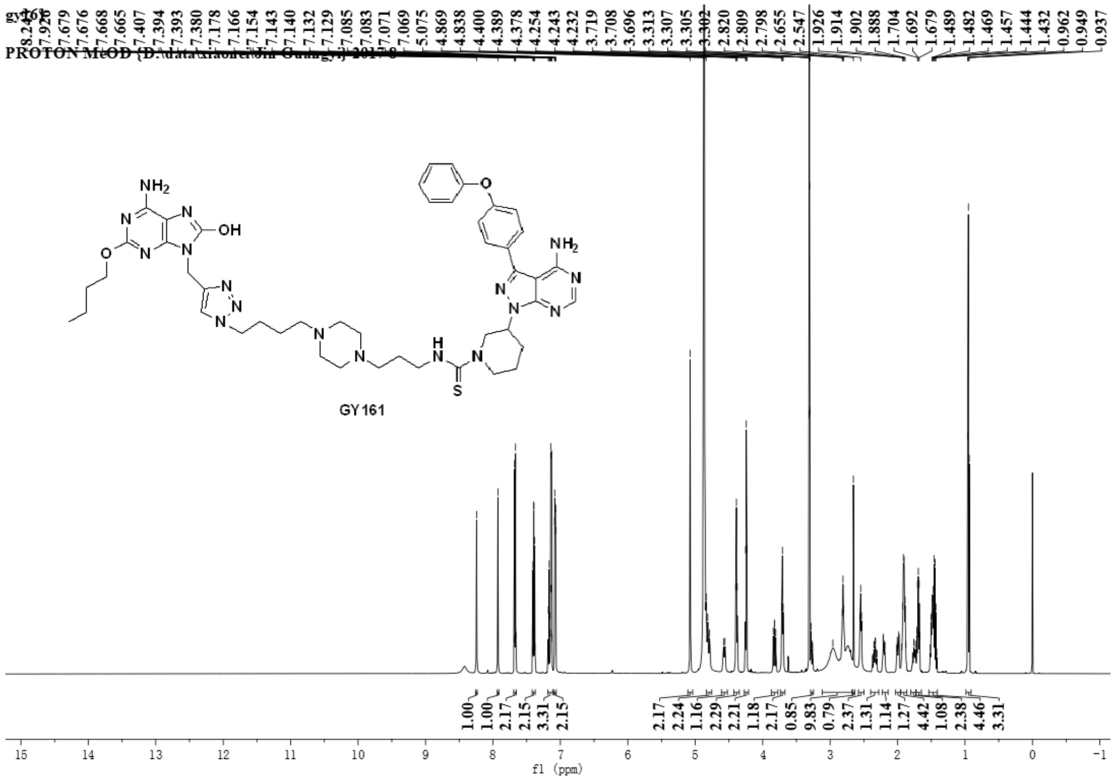

[1] H NMR of **GY161** (600 MHz, CD_3OD)

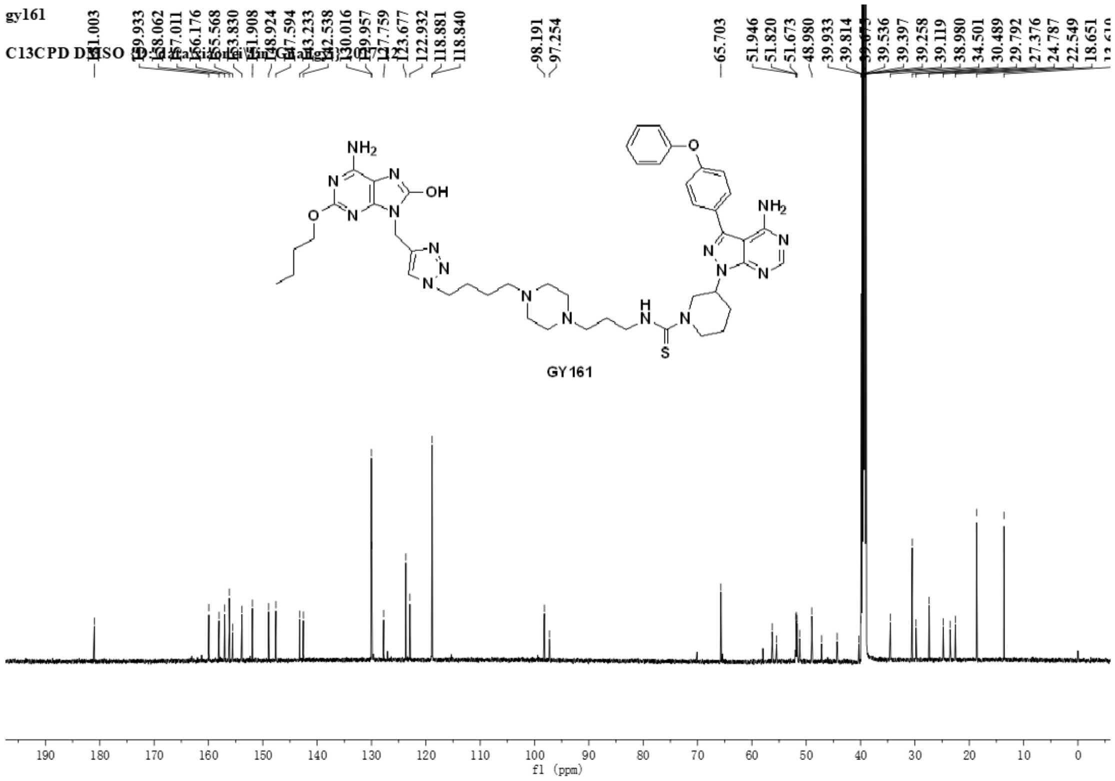

[13] C NMR of **GY161** (151 MHz, DMSO-d_6)

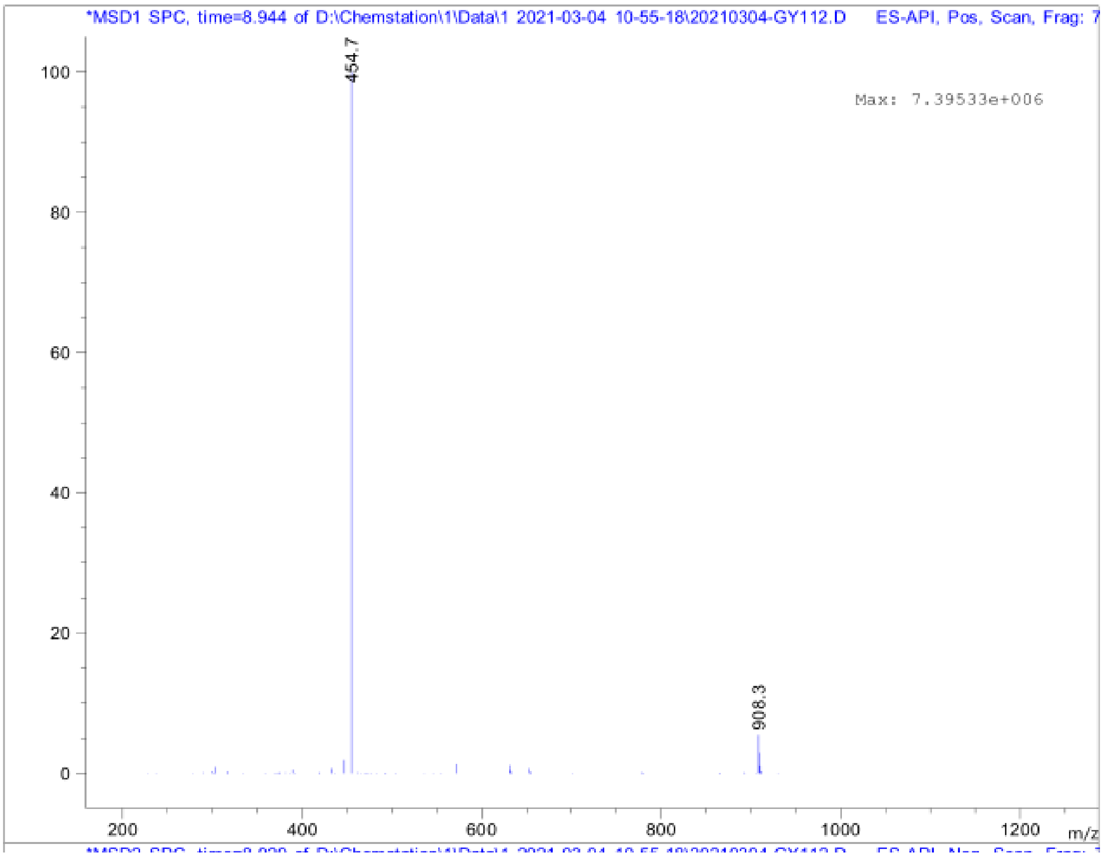

ESI-MS spectra of GY112 (908.3 $[M+H]^+$)

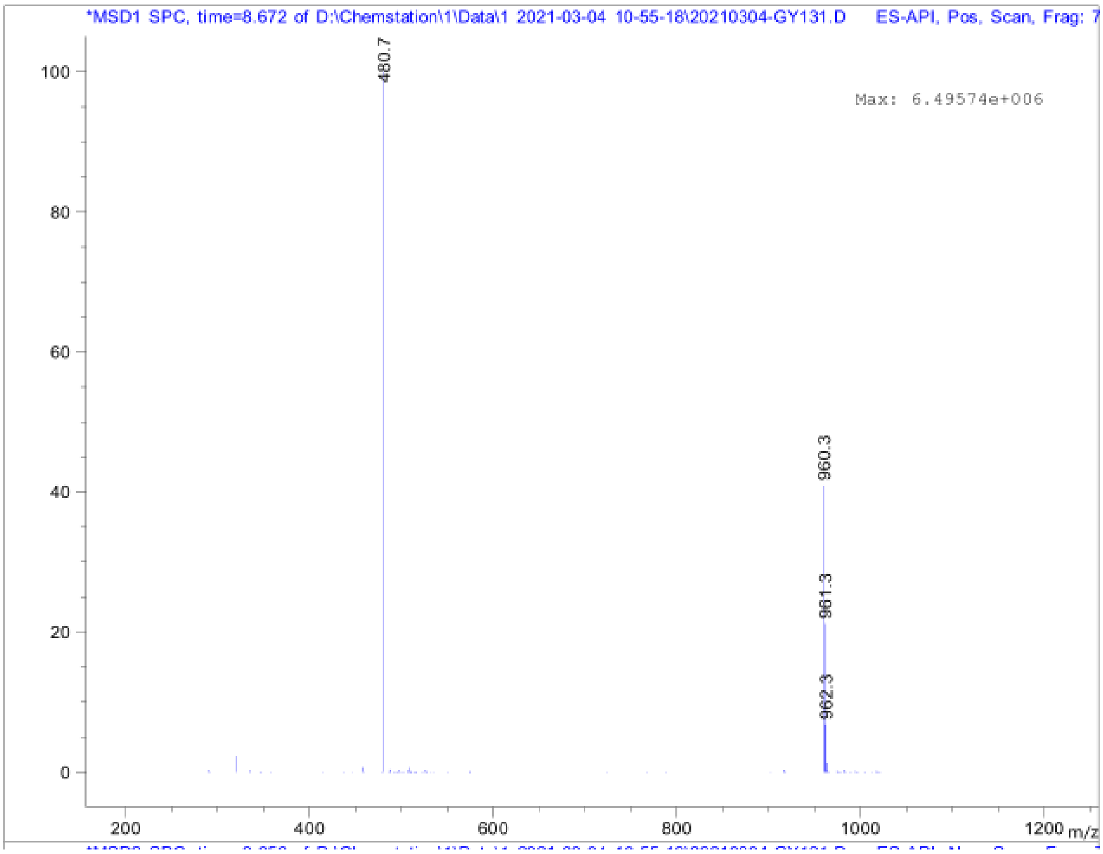

ESI-MS spectra of GY131 (960.3 $[M+H]^+$)

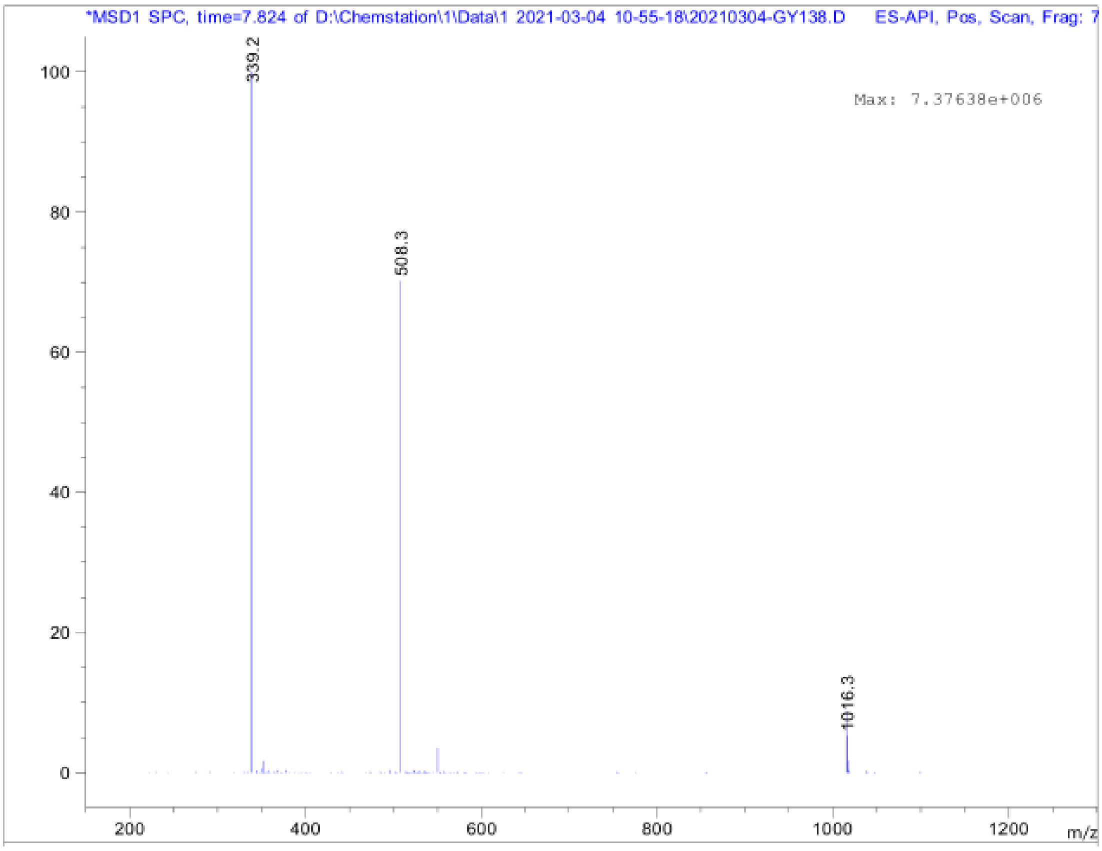

ESI-MS spectra of GY138 (1016.3 $[M+H]^+$)

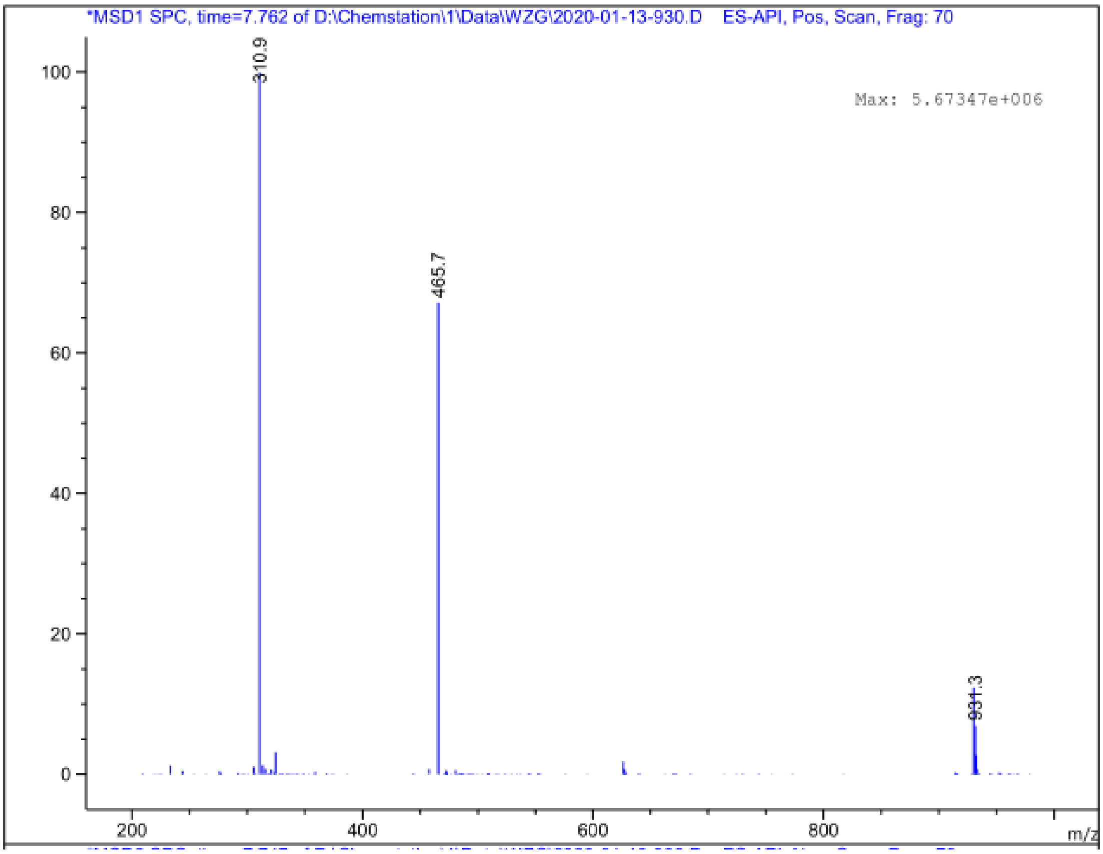

ESI-MS spectra of GY161 (931.3 $[M+H]^+$)

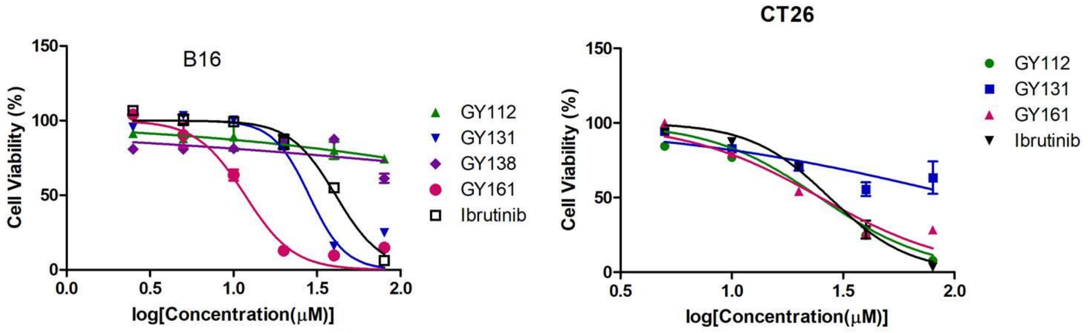

Figure S2. Antiproliferative effects of conjugates of ibutinib with GY100. B16 and CT26 cells were incubated with serial dilutions of compounds at concentration ranging form 2.5–80.0 μM for 48 h.

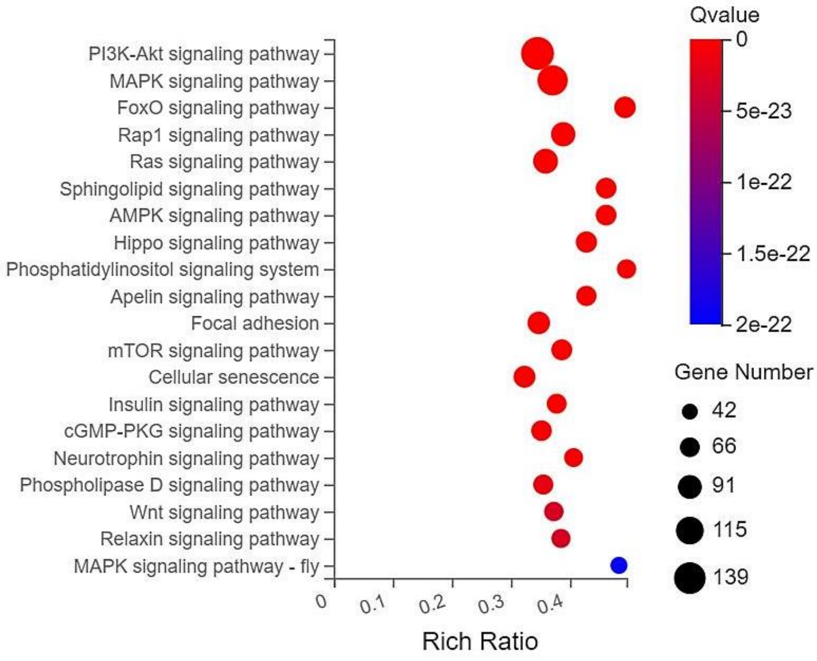

Figure S3. The top 20 pathways involved in B16 cells after GY161 treatment for 24 h compared with the contro1 (DMSO treated).

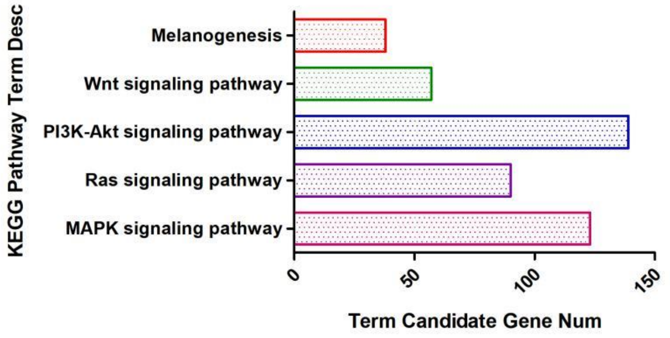

Figure S4. Some major differentially pathways in KEGG Pathway analysis.

Table S1. Expression levels of some major differentially genes in KEGG Pathway analysis.

st_gene_id	gene_id	gene_symbol	fpkm_Control	fpkm_GY161
G10090_7100	17295	Met	148.91	43.7
G10090_937	18707	Pik3cd	6.84	3.61
G10090_5175	18710	Pik3r3	49.00	15.68
G10090_13427	18709	Pik3r2	33.03	20.48
G10090_12466	11651	Akt1	401.54	235.76
G10090_13353	12387	Ctnnb1	446.30	215.62
G10090_748	17342	Mitf	100.20	16.09
G10090_6495	22173	Tyr	63.33	29.79
G10090_2153	22178	Tyrp1	5095.32	3267.94
G10090_23351	13190	Dct	3692.73	1846.72
